# Validation of Recombinant Type I Interferon Antiviral Activity Against Porcine Epidemic Diarrhea Virus In Vitro and In Vivo

**DOI:** 10.3390/vetsci13030249

**Published:** 2026-03-06

**Authors:** Luyu Du, Ruili Zhang, Shuyang Wang, Shanshan Han, Shuyu Zhang, Fanliang Meng, Zheng Fang, Xinyuan Wang, Rui Zhao, Ronglian Dai, Liting Qin, Chuang Lyu, Gang Wang

**Affiliations:** 1 Qingdao Jiazhi Biotechnology Co., Ltd., Qingdao 266100, China; ddlyuuu@163.com (L.D.); zhangruilichina@126.com (R.Z.); wsy8450@163.com (S.W.); hsshyr@126.com (S.H.); dairl@newhope.cn (R.D.); 2 Shandong Provincial Key Laboratory of Zoonoses, College of Veterinary Medicine, Shandong Agricultural University, Taian 271018, China; zsy81003@gmail.com (S.Z.); 18754875921@163.com (F.M.); 18005490835@163.com (X.W.); 13281339753@163.com (R.Z.)

**Keywords:** PEDV, PED, IFN-α, antiviral activity

## Abstract

Porcine epidemic diarrhea virus (PEDV), a coronavirus responsible for severe watery diarrhea in neonatal piglets, leads to significant economic losses globally. Effective prevention and treatment of PEDV infection is critical to the swine industry. Currently, there are no available therapeutic drugs for PEDV. Porcine interferons (poIFNs) have been identified as promising molecules against a series of swine viruses due to their broad-spectrum antiviral and immunomodulatory properties. In this study, we demonstrated that type I interferon-α (IFN-α) offered both prophylactic and therapeutic benefits against PEDV infection. Recombinant poIFN-α produced by a prokaryotic expression system was purified through affinity chromatography, and its prophylactic and therapeutic effects against PEDV infection were evaluated in vitro and in vivo through RT-qPCR, clinical symptom monitoring, and pathological examination. In vitro studies revealed a strong antiviral activity of poIFN-α against PEDV in Vero E6 cells, with a more pronounced prophylactic effect compared to therapeutic outcomes. In vivo studies showed that poIFN-α significantly alleviated clinical diarrhea in PEDV-infected piglets and reduced intestinal viral loads. These findings suggest that poIFN-α holds considerable promise as an antiviral agent for PEDV and provides a foundation for the development of therapeutic strategies targeting this virus.

## Simple Summary

Porcine epidemic diarrhea virus currently causes significant economic losses to the global swine industry. By exploring the antiviral potential of porcine interferon-alpha against porcine epidemic diarrhea virus, this study aims to develop effective preventive and therapeutic measures. Evaluations using cell experiments and piglet infection models revealed that porcine interferon-alpha exhibits potent antiviral activity at the cellular level, with prophylactic effects being superior to therapeutic outcomes. In virus-infected piglets, it significantly alleviated diarrhea symptoms and reduced intestinal viral loads. The research findings confirm that porcine interferon-alpha holds promise as an effective antiviral agent against porcine epidemic diarrhea virus. This not only provides a foundation for new treatment strategies to control the virus but also helps reduce agricultural economic losses, carrying clear social significance for maintaining the stable development of the livestock industry.

## 1. Introduction

Interferons (IFNs) are a class of proteins with diverse biological activities, including antiviral, immunomodulatory, and antitumor. Among them, type I IFN (IFN-I) subtypes, e.g., IFN-α and IFN-β, are the most well-characterized ones that are primarily produced by virus-infected cells, exhibiting broad-spectrum antiviral activities. Host cells respond rapidly to viral infections through the secretion of IFN-α [1]. The JAK-STAT signaling pathway is activated after binding of IFNs to their specific receptor, and then hundreds of antiviral proteins are expressed in cells, which results in inhibition of viral replication, translation, or spread [2]. Additionally, IFN-α exerts immunomodulatory effects by enhancing immune cell activity and promoting immune responses [3]. In recent years, IFN has gained significant attention in biomedical research as a versatile antiviral biopharmaceutical candidate. Advances in genetic engineering have facilitated the production of recombinant IFN-α from various species using both prokaryotic and eukaryotic expression systems [4,5]. The efficacy of recombinant IFN-α in treating SARS-CoV-2 infections has been well-documented previously [6,7,8], highlighting promising therapeutic potential against viral diseases. Recently, a large body of research has focused on investigating the structural characteristics, classification, functional properties, and biological mechanisms of porcine IFN (poIFN). The potent antiviral activity and clinical efficacy of poIFN are increasingly recognized. The poIFN of low dose has been demonstrated to elicit strong antiviral and immunomodulatory responses [1,9]. Given these findings, poIFN is expected to play a pivotal role in the prevention and control of porcine viral diseases.

Porcine epidemic diarrhea virus (PEDV), a member of the Alphacoronavirus genus within the family *Coronaviridae*, is a single-stranded, positive-sense RNA virus [10]. Porcine epidemic diarrhea (PED) caused by PEDV is an acute and highly contagious enteric disease that occurs year-round, with higher incidence rates in winter and spring. The virus is transmitted via the fecal–oral route and clinically manifests as severe watery diarrhea, vomiting, dehydration, and extremely high mortality rates in neonatal piglets [11,12,13]. The disease primarily causes intestinal damage, including atrophy of small intestinal villi, destruction of intestinal epithelial cells, and thinning of the intestinal wall [14]. PED results in substantial neonatal piglet death, declines in production performance, and increased prevention and control costs, severely hindering the recovery of production capacity, which has significant economic repercussions for the swine industry. To date, there have been no fully effective therapeutic agents or vaccines for controlling PEDV infection, and existing vaccines and treatment strategies face numerous challenges in preventing PEDV infection [15]. Conventional vaccines mainly induce systemic IgG, which fails to neutralize the virus within the intestines [16]. Furthermore, most of the commercial vaccines do not stimulate the production of sufficient secretory IgA (sIgA), which is taken as the most important antibody subtype providing immunoprotection gained from sows for piglets [17,18]. The rapid mutation of PEDV also results in antigenic mismatches between vaccine strains and circulating field strains, reducing vaccine efficacy. These challenges have hindered effective control of PED.

Considering that IFNs exert antiviral effects through multiple mechanisms, establishing a robust antiviral defense system that inhibits viral replication and transmission, the development of novel recombinant IFNs and research on their preventive and therapeutic effects against viral diseases have become increasingly critical. This study aims to evaluate the prophylactic and therapeutic effects of recombinant poIFN-α against PEDV infection, providing experimental evidence for the development of novel antiviral strategies. To this end, recombinant poIFN-α was successfully expressed and purified. Subsequently, both in vitro and in vivo experiments were designed and conducted to assess the preventive and therapeutic efficacy of the recombinant poIFN-α against PEDV. The results consistently demonstrated that recombinant poIFN-α displayed significant preventive and therapeutic effects against PEDV. These findings not only provide a solid theoretical foundation for the future development of antiviral therapeutics but also offer valuable insights into strategies for the prevention and control of PED.

## 2. Materials and Methods

### 2.1. Cell Culture and Virus Inoculation

Vero E6 cells (kept by Qingdao Jiazhi Biotechnology Co., Ltd, Qingdao, China.) were cultured in DMEM (Gibco, Waltham, MA, USA) supplemented with 10% (*v*/*v*) fetal bovine serum (Nulen Biotech, Shanghai, Chian) and 1% penicillin–streptomycin at 37 °C with 5% CO_2_. The GII PEDV WF2024 (isolation and kept by Qingdao Jiazhi Biotechnology Co., Ltd.) was mixed with DMEM containing 10 µg/mL EDTA-free trypsin (Gibco, USA) for inoculation onto Vero E6 cells. When the cells exhibited syncytia, the supernatant after three cycles of freeze–thaw and centrifugation was collected as viral stocks and stored at −80 °C in a freezer. All subsequent experiments used viral stocks prepared from the PEDV WF2024 strain.

### 2.2. Preparation of Recombinant poIFN-α

Primers listed in Table 1 were used to amplify full-length CDS of the poIFN-α gene (GenBank: AB369102.1). The collected PCR product and the pET32b (+) vector (Vazyme, Nanjing, China) were separately digested with *Bam* HI and *Hin* dIII (TaKaRa, Qingdao, China) restriction enzymes. The digested fragments were ligated, and then the chosen monoclonal constructs were subjected to sequencing to verify the correct construction of the recombinant plasmid, followed by transforming into Rossetta™ (DE3) competent cells (Solarbio, Beijing, China). The transformed cells were induced with 0.6 mM IPTG and cultured at 37 °C for 6 h. Samples were collected for SDS-PAGE analysis. After centrifugation of the lysate, the pellet was resuspended in inclusion body equilibration buffer and incubated overnight at 4 °C. The denatured protein was centrifuged to collect the supernatant, which was purified by using Ni^2+^-NTA resin, eluting with different concentrations of imidazole, followed by refolding. The protein was slowly added to the refolding dilution buffer, incubated at 4 °C for 24 h, then transferred into a dialysis bag for slow stirring dialysis at 4 °C, 300 rpm to allow refolding. After refolding, Triton X-114 was employed to remove endotoxin, followed by concentration for Western blot analysis. The concentration of the purified protein was determined by establishing a standard curve using the BCA protein concentration assay kit (Beyotime, Shanghai, China).

### 2.3. Anti-VSV Activity Assay for poIFN-α Protein

PK-15 cells (kept by Qingdao Jiazhi Biotechnology Co., Ltd.) were cultured in DMEM (Gibco, USA) supplemented with 10% (*v*/*v*) fetal bovine serum (Nulen Biotech, Shanghai, China) and 1% penicillin–streptomycin, seeded in 96-well plates, and incubated at 37 °C with 5% CO_2_. The cells were treated with 10-fold serial dilutions of poIFN-α (100 µL/well, 8 replicates per dilution). After 24 h incubation, cells were challenged with Vesicular Stomatitis Virus (VSV) (kept by Qingdao Jiazhi Biotechnology Co., Ltd.) at 100 TCID_50_. Cytopathic effects (CPEs) were observed for 48 h, and antiviral activity was calculated using the Reed–Muench method.

### 2.4. Western Blot

The purified, refolded, and concentrated protein was mixed with loading buffer and denatured by boiling. Protein separation was carried out using 12% SDS-PAGE. After separation, the protein was transferred to a PVDF membrane (Millipore, Billerica, MA, USA) and blocked with 5% skim milk. Detection was performed using an anti-His-tag antibody (mouse monoclonal) (1:1000, Beyotime, Shanghai, China) as the primary antibody and HRP-conjugated goat anti-mouse IgG (H + L) (1:1000, Beyotime, China) as the secondary antibody.

### 2.5. In Vitro Evaluation of the Effect of poIFN-α on PEDV Replication

Vero E6 cells seeded into six-well plates (NEST, Wuxi, China) were cultured until 90% confluency. To evaluate the preventative use of poIFN-α in vitro, the cells in each well were treated with 10^7^ IU poIFN-α for 6 h, followed by an inoculation with 10^4^ TCID_50_ PEDV. Cell samples were collected at 2, 4, 6, 8, 12, 24, and 36 h post-infection (hpi) to assess the effect of poIFN-α against PEDV. To evaluate the therapeutic use of poIFN-α in vitro, the cells in each well were infected with 10^4^ TCID_50_ PEDV, then treated with 10^7^ IU poIFN-α. Cell samples were collected at 2, 4, 6, 8, 12, 24, and 36 hpi to assess the effect of poIFN-α against PEDV. CPEs were observed at 2, 4, 6, 8, 12, 24, and 36 hpi, and viral RNA copy numbers were quantified using a real-time RT-qPCR kit (Qingdao Jiazhi Biotechnology Co., Ltd., China).

### 2.6. In Vivo Evaluation of the Effect of poIFN-α on PEDV Replication

#### 2.6.1. Animal Experiment Design

A total of 9 twenty-day-old healthy piglets were used in the animal experiment. The animals were randomly assigned to three groups: (1) experimental group (PEDV + poIFN-α); (2) control group (PEDV only); and (3) negative control group (MOCK). Both experimental and control groups were orally administered 5 mL of PEDV suspension with a titer of 10^4^ TCID_50_/mL (A challenge dose of 10^4^ TCID_50_ was predetermined via pilot studies to induce reliable viral replication and typical diarrhea without excessive mortality). The experimental group began receiving poIFN-α pretreatment two days prior to viral challenge. The poIFN-α was diluted at a 1:100 ratio and administered orally once daily at a dose of 1 mL (drug concentration: 10^5^ IU). This same regimen was continued for four consecutive days following the challenge. The MOCK group received an equal volume of DMEM. The animal experiments were approved by the Chinese Regulations for Laboratory Animals and Animal Ethics Committee of Shandong Agricultural University, China (approval number SDAUA-2025-089).

#### 2.6.2. Clinical Evaluation

Clinical signs in piglets were monitored and recorded every 24 h, and daily changes were measured at the same time each day using an electronic scale (Kaifeng, China) and a thermometer (Jinfengyuan, China). Fecal condition was evaluated and scored as follows: 0 points for normal, 1 point for mild diarrhea, 2 points for moderate diarrhea, and 3 points for severe diarrhea. Normal feces were solid; mild diarrhea was semi-solid; moderate diarrhea consisted of pasty feces with fecal adhesion around the anus; severe diarrhea was characterized by watery stools, accompanied by vomiting and large amounts of watery feces around the anus.

#### 2.6.3. Histopathological Examination and Immunohistochemistry

At the end of the experiment, all piglets in the three groups were euthanized and necropsied to observe the pathological changes in the intestines. The tissues derived from the duodenum, jejunum, and ileum were collected for the subsequent analysis. The intestinal tissues were fixed in formalin and then processed through washing, dehydration, and clarification, followed by embedding in paraffin. The tissues were then sectioned onto slides (Solarbio, Beijing, China), stained with hematoxylin and eosin (HE), and examined under a microscope (NEXCOPE, Ningbo, China). For immunohistochemical (IHC) evaluation of viral antigens in the tissue sections, a rabbit anti-PEDV-N protein monoclonal antibody was used as the primary antibody, and HRP-conjugated goat anti-rabbit IgG (Beyotime, Shanghai, China) was used as the secondary antibody. The sections were visualized using a DAB substrate kit (Solarbio, China).

#### 2.6.4. Detection of Viral Loads

The pMD19T-PEDV-N plasmid (provided by Qingdao Jiazhi Biotechnology Co., Ltd.) was serially diluted to concentrations ranging from 10^1^ to 10^10^ copies/μL. A standard curve was constructed using more than five points, with the logarithmic values of the standard plasmid copy number concentrations as the x-axis and the Ct values as the y-axis to obtain the linear equation. Anal swabs were submerged in 500 μL of saline and stored at −80 °C freezer followed by RNA extraction using an RNA extraction kit (Qingdao Jiazhi Biotechnology Co., Ltd., China), and the viral RNA copy number was determined by quantitative detection using a real-time RT-qPCR kit (Qingdao Jiazhi Biotechnology Co., Ltd., China) with the following cycling conditions: 52 °C for 10 min (reverse transcription), 95 °C for 30 s (pre-denaturation), followed by 40 cycles of 95 °C for 10 s and 60 °C for 30 s (fluorescence collection).

### 2.7. Statistical Analyses

Statistical analyses were performed using GraphPad Prism 8 software. Data are presented as mean ± standard deviation (SD) or standard error of the mean (SEM) from three independent experiments. One-way analysis of variance (ANOVA) with Tukey’s multiple comparison test was used to assess differences among multiple groups. with Student’s *t*-test applied for pairwise comparisons. Throughout the manuscript, *p* < 0.05 was used as the criterion for statistical significance (* *p* < 0.05, ** *p* < 0.01, and *** *p* < 0.001, ns: no statistical significance).

## 3. Results

### 3.1. Prokaryotic Expression of Recombinant PoIFN-α

**Figure 1:**
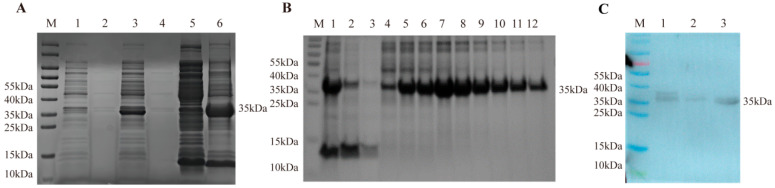
Prokaryotic expression and purification of recombinant PoIFN-α. (**A**) Induction expression of poIFN-α. Lane M: Marker; Lane 1: Pre-induction bacterial culture; Lane 2: Post-induction supernatant; Lane 3: Post-induction precipitate; Lane 4: Post-induction supernatant; Lane 5: Supernatant after sonication; Lane 6: Precipitate after sonication. (**B**) Purification of porcine IFN-α without using imidazole for elution. Lane M: Marker; Lane 1: Precipitate before purification; Lane 2: Flow-through fraction; Lane 3: Equilibration buffer; Lanes 4–12: 50 mM imidazole solution; 80 mM imidazole solution; 100 mM imidazole solution; 150 mM imidazole solution; 200 mM imidazole solution; 250 mM imidazole solution; 300 mM imidazole solution; 400 mM imidazole solution; 500 mM imidazole solution. (**C**) Identification of recombinantly expressed poIFN-α protein by Western blot. Lane M: Marker; Lane 1: Purified poIFN-α; Lane 2: Refolded poIFN-α; Lane 3: Concentrated poIFN-α (the original pictures can be found in Figure S1) .

The construction of recombinant plasmid pET32b (+)—poIFN-α was verified by restriction enzyme digestion and sequencing. Following IPTG induction, the target protein was expressed. SDS-PAGE analysis for the collected supernatant and pellet post-ultrasonic disruption showed the presence of a potential target protein at around 35 kDa in the form of inclusion bodies (Figure 1A). Inclusion body proteins were eluted under different concentrations of imidazole, and better purification was achieved with the elution buffer containing 150 mM imidazole. The protein concentration was determined to be 3.7 mg/mL (Figure 1B). After endotoxin removal, the endotoxin level was measured to be 310 EU/mL. The expressed recombinant protein poIFN-α-His was further identified by Western blot using anti-His monoclonal antibody. After purification, refolding, and concentration, a distinct protein band appeared at 35 kDa (Figure 1C). The antiviral activity against VSV was determined to be 10^7^ IU/mg.

### 3.2. In Vitro Protective Effect of poIFN-α

**Figure 2:**
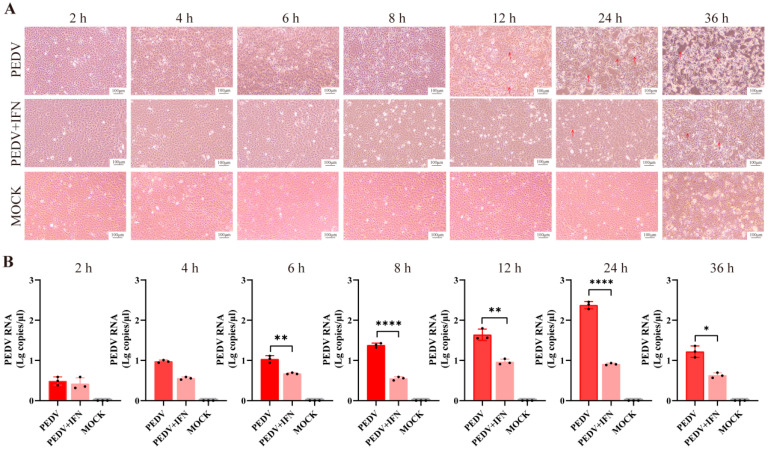
Protective effect of poIFN-α against PEDV infection in Vero E6 cells. Vero E6 cells were treated with 10^7^ IU poIFN-α. (**A**) CPEs in Vero E6 cells at different time points post-infection (2, 4, 6, 8, 12, 24, and 36 hpi). Red arrows indicate areas of significant cellular damage or changes. The scale bar represents 100 μm. (**B**) RT-qPCR results of PEDV copies in cells at different time points post-infection (2, 4, 6, 8, 12, 24, and 36 hpi). Data are presented as Mean ± SD (*n* = 3). * *p* < 0.05, ** *p* < 0.01, **** *p* < 0.0001, compared to the corresponding PEDV group at the same time point (the original pictures can be found in Figure S2) .

CPEs were monitored at various time points post-PEDV infection. In the PEDV group, cells exhibited increased intercellular space since 12 hpi, with syncytia formation becoming evident as the infection progressed, as indicated by red arrows (Figure 2A). In contrast, cells in the PEDV + IFN group showed increased intercellular space since 24 hpi. Furthermore, at 12, 24, and 36 hpi, the CPEs in the PEDV + IFN group were significantly milder than the PEDV group (Figure 2A, red arrows). Viral load measurements revealed significant differences between the PEDV and PEDV + IFN groups since 6 hpi (*p* < 0.01). At 8 and 24 hpi, the viral loads in the PEDV + IFN group were significantly lower than those in the PEDV group, with the most pronounced differences observed at 24 hpi (*p* < 0.001) (Figure 2B).

### 3.3. In Vitro Therapeutic Effect of poIFN-α

**Figure 3:**
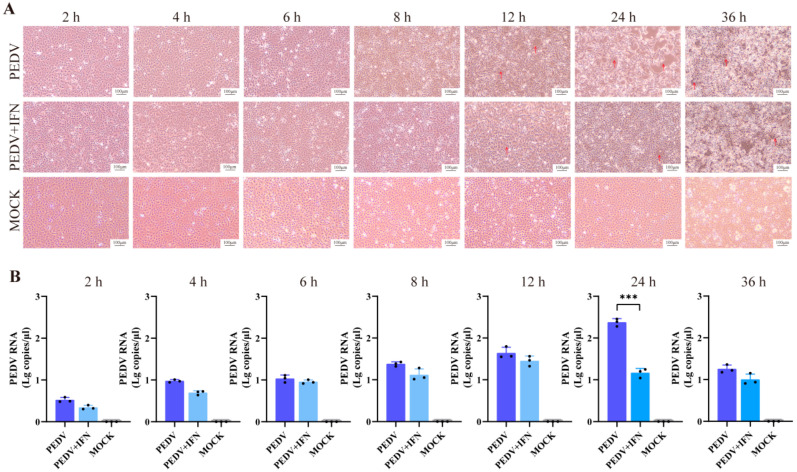
Therapeutic effect of poIFN-α against PEDV infection in Vero E6 cells. Vero E6 Cells were treated with 10^7^ IU poIFN-α after PEDV infection. (**A**) CPEs in Vero E6 cells at different time points post-infection (2, 4, 6, 8, 12, 24, and 36 hpi). Red arrows indicate areas of significant cell damage or changes. The scale bar represents 100 μm. (**B**) RT-qPCR results of PEDV copies in cells at different time points post-infection (2, 4, 6, 8, 12, 24, and 36 hpi). Data are presented as Means ± SD (*n* = 3). *** *p* < 0.001, compared to the corresponding PEDV group at the same time point.

CPEs were assessed at various time points post-infection. In the PEDV group, cells showed increased intercellular space since 12 hpi, with syncytia formation becoming evident as the infection progressed (Figure 3A, red arrows). In contrast, cells in the PEDV + IFN group showed increased intercellular space since 12 hpi. However, at 12, 24, and 36 hpi, the degree of CPEs in the PEDV + IFN group was significantly milder than that in the PEDV group (Figure 3A, red arrows). Viral load analysis at 24 hpi revealed a significant difference between PEDV and PEDV + IFN groups (*p* < 0.001) (Figure 3B).

### 3.4. PoIFN-α Improves the Growth Performance of PEDV-Infected Piglets

**Figure 4:**
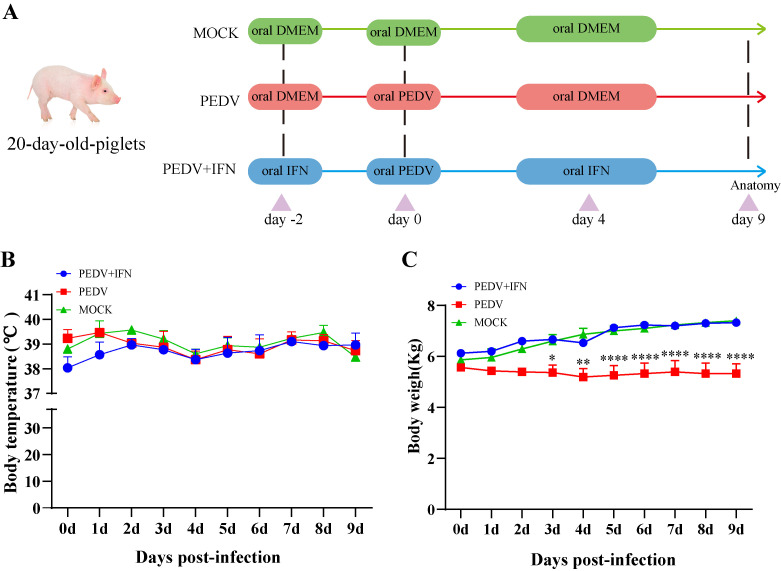
PoIFN-α improves the growth performance of PEDV-infected piglets. (**A**) Schematic diagram for assessing the effect of poIFN-α on the prevention and control of PEDV infection in three groups of piglets. MOCK group (green line), PEDV group (red line), and PEDV + IFN group (blue line). (**B**) Changes in body temperature of piglets in each group. Data are presented as Means ± SEM (*n* = 3). (**C**) Changes in body weight of piglets in each group. Data are presented as Means ± SEM (*n* = 3). * *p* < 0.05, ** *p* < 0.01, **** *p* < 0.0001, compared to the corresponding MOCK group at the same time point.

To evaluate the efficacy of poIFN-α in preventing PEDV infection, piglets received daily oral administration of poIFN-α for two days prior to PEDV challenge and continued until 4 days post-infection (dpi). Clinical signs were monitored daily, and the rectal swab samples were collected for analysis (Figure 4A). The results showed that body temperatures remained stable across all groups (Figure 4B). The PEDV group experienced a decrease in body weight since day 1 post-challenge, reaching the lowest point on day 4, which was significantly different from the MOCK group (*p* < 0.0001). In contrast, the PEDV + IFN group showed only a slight weight decrease on 3 dpi, followed by continuous weight gain starting from the next day (Figure 4C).

### 3.5. PoIFN-α Reduces the Incidence of Diarrhea in Piglets

**Figure 5:**
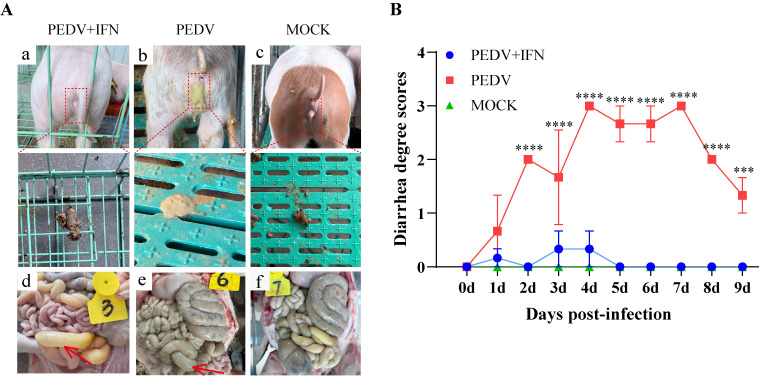
PoIFN-α reduces the incidence of diarrhea in piglets. (**A**) Clinical observations of piglets in each group. (**a**–**c**): severity of diarrhea in piglets; (**d**–**f**): macroscopic pathological changes in the small intestine of piglets, and red arrows indicate the areas of lesions. (**a**,**d**): PEDV + IFN group; (**b**,**e**): PEDV group; (**c**,**f**): MOCK group. (**B**) Fecal score for each group of piglets. Data are presented as Means ± SEM (*n* = 3). *** *p* < 0.001, **** *p* < 0.0001, compared to the corresponding MOCK group at the same time point.

In the PEDV group, the clinical signs watery diarrhea, lethargy, and dehydration were observed since day 1 post-challenge, and adherent feces were seen around the anus (Figure 5A(b)). In contrast, in the PEDV + IFN group, no significant diarrhea was observed, manifesting normal feces and perianal regions (Figure 5A(a)). Although some individuals in the PEDV + IFN group exhibited mild diarrhea, probably due to individual variation in the poIFN-α response, the symptoms were transient and recovered within one day. In the MOCK group, the piglets displayed no diarrhea or other adverse clinical signs (Figure 5A(c)). Post-mortem examinations revealed pathological changes in the dissected jejunum and ileum of piglets in both PEDV and PEDV + IFN groups, including transparent, thin intestinal walls and intestinal dilation, as indicated by red arrows (Figure 5A(d,e)). The severity of diarrhea, as scored in piglets, was consistently lower in the PEDV + IFN group compared to that in the PEDV group. In comparison with the MOCK group, there was a significant difference (*p* < 0.0001) (Figure 5B).

### 3.6. The Antiviral Activity of poIFN-α in PEDV-Infected Piglets

**Figure 6:**
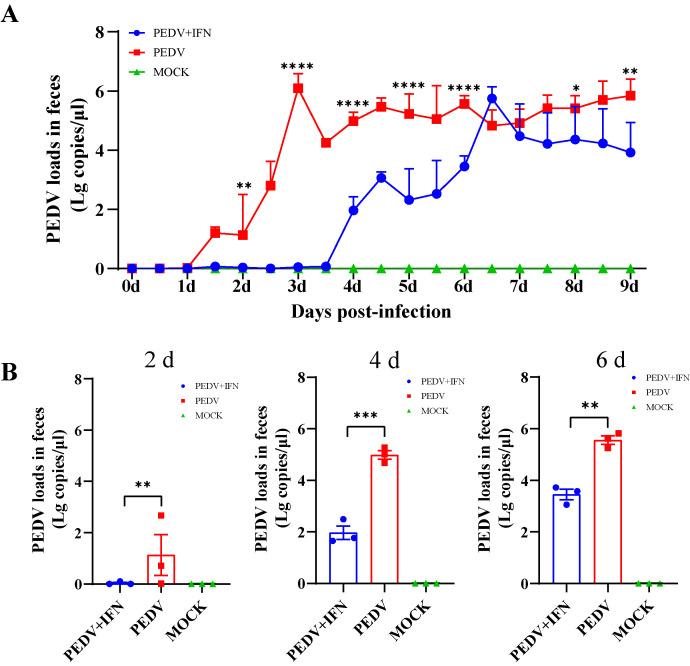
Effect of poIFN-α on viral loads post-PEDV challenge in piglets. (**A**) The trend in PEDV loads from 2 to 8 dpi. Data are presented as Means ± SEM (*n* = 3). (**B**) PEDV loads in anal swabs at 2, 4, and 6 dpi, respectively. Data are presented as Means ± SEM (*n* = 3). * *p* < 0.05, ** *p* < 0.01, *** *p* < 0.001, **** *p* < 0.0001, compared to the corresponding PEDV group at the same time point.

Real-time qRT-PCR was performed to assess PEDV RNA levels in anal swab samples collected from the experimental piglets. In the PEDV group, viral loads began to increase gradually from day 1 post-challenge. In contrast, in the PEDV + IFN group, viral loads remained low during the first three days and then began to rise. Following the discontinuation of poIFN-α, viral loads in the PEDV + IFN group increased gradually with the progression of days but remained lower than those in the PEDV group (Figure 6A). The viral loads at 2, 4, and 6 dpi were consistently significantly lower in the PEDV + IFN group compared to those in the PEDV group (Figure 6B).

### 3.7. The Effect of poIFN-α on Intestinal Damage in PEDV-Infected Piglets

**Figure 7:**
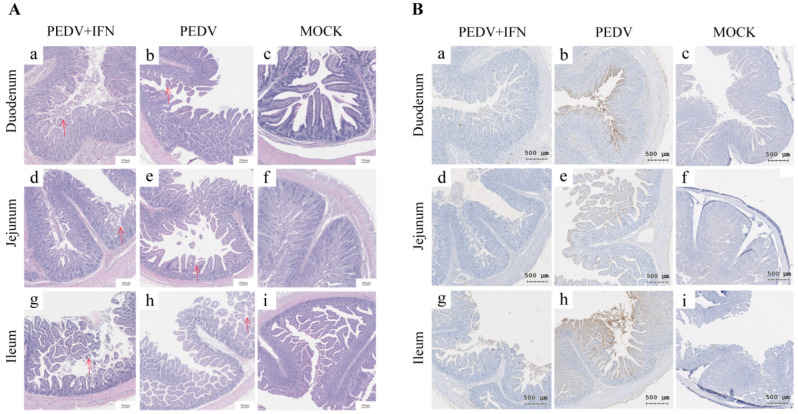
The Effect of poIFN-α on intestinal damage post-PEDV challenge in piglets. (**A**) HE staining of small intestine tissue sections. Arrows indicate pathological changes (Scale bar: 200 μm). (**B**) IHC staining of small intestine tissue.

Histological analysis revealed mild villous atrophy and crypt hyperplasia of duodenal tissue in the PEDV + IFN group (Figure 7A(a)). In the jejunum, villous atrophy and hyperplasia were more pronounced (Figure 7A(d)), while the villous structure in the ileum remained largely intact (Figure 7A(g)). In contrast, the tissue in the PEDV group displayed significant villous atrophy and crypt hyperplasia in the duodenum, with a marked reduction in villous height (Figure 7A(b)). Both jejunum and ileum exhibited severe villous atrophy and prominent crypt hyperplasia caused by PEDV infection (Figure 7A(e,h)).

IHC using specific antibodies against PEDV revealed widespread distribution of PEDV antigens in the intestinal villi from the PEDV group (Figure 7B(b,e,h)). In the PEDV + IFN group, the detected PEDV antigens and distribution were significantly reduced compared to those in the PEDV group (Figure 7B(a,d,g) vs. (b,e,h)). No PEDV antigens were detected in the intestinal tissues from the MOCK group (Figure 7B(c,f,i)).

## 4. Discussion

Since the emergence of PEDV variants in China in 2010, the virus has rapidly spread across the country, causing high mortality in piglets and substantial economic losses to the swine industry [12]. Despite the availability of various drugs and vaccines in recent years, the rapid mutation rate of PEDV has significantly reduced the effectiveness of existing therapeutic and preventive measures [13,19]. Currently, no fully effective commercial vaccines for controlling PED [20,21]. In this study, the preventive and therapeutic efficacy of recombinant poIFN-α against PEDV was evaluated both in vitro and in vivo, demonstrating promising potential for its clinical application. The recombinant poIFN-α was successfully expressed in the form of inclusion bodies. After optimized refolding under purified conditions, the protein regained antiviral activity. These results support the feasibility of large-scale production and application of recombinant poIFN-α, establishing a theoretical foundation for the development of cost-effective antiviral therapies.

The preventive and therapeutic efficacy of the recombinant poIFN-α against PEDV was assessed both in vitro and in vivo. In vitro results showed that poIFN-α significantly alleviated the CPEs induced by PEDV, delayed the onset of CPEs, and substantially reduced viral loads. Further analysis revealed that prophylactic administration exhibited superior efficacy compared to therapeutic administration. Prophylactic interferon administration in vivo establishes an antiviral state, pre-activates innate immune responses, and builds antiviral defense barriers, thereby effectively ameliorating disease clinical manifestations [22]. These findings align with previous studies demonstrating the enhanced effectiveness of prophylactic treatment in inhibiting viral replication [23]. This can be attributed to prophylactic treatment activating the host antiviral immune response prior to viral infection, thereby enhancing host antiviral capacity and more effectively inhibiting viral replication and spread. In contrast, therapeutic administration occurs after substantial viral replication and dissemination, at which point extensive cellular damage and high viral loads may impair the antiviral immune activation by poIFN-α, therefore reducing therapeutic efficacy [24,25].

In vivo experiments were further performed to comprehensively evaluate the protective effects of poIFN-α on intestinal function in PEDV-infected piglets. The results indicated that poIFN-α positively impacted growth performance. Although poIFN-α alleviated clinical signs to some extent, mild histopathological lesions were still observed in tissue sections. As noted in prior studies by Song et al., once damage to the small intestinal mucosa occurs, inhibiting further viral replication alone may not be sufficient to fully restore villous integrity [26]. This highlights the importance of early intervention in minimizing intestinal tissue damage. The present study further confirms the protective efficacy of poIFN-α in a PEDV-infected animal model, demonstrating its beneficial effects in mitigating early-stage PEDV infection in piglets.

This study primarily aimed to evaluate the clinical outcomes and virological parameters in PEDV-infected piglets following poIFN-α administration. PEDV infection in 20-day-old piglets induces clinical signs of diarrhea with low mortality, which meets the requirements for poIFN-α antiviral experiments; in contrast, infection in piglets under 10 days of age results in high mortality. Building upon the established viral model in 20-day-old piglets, we will further investigate the efficacy of poIFN-α in younger piglets. However, to fully elucidate the clinical potential of poIFN-α and optimize its application strategies, the following aspects warrant further in-depth investigation. First, the small sample size (*n* = 3) necessitates larger-scale studies to more accurately evaluate efficacy and integrate dedicated safety assessment protocols to holistically evaluate the safety profile and clinical applicability of poIFN-α therapy. Additionally, only a single administration dose (10^5^ IU/mL) was explored, and further research is needed to determine the optimal dosage and regimen. Lastly, while the orally administered poIFN-α was not encapsulated and may be subject to degradation, oral interferon administration has proven effective in both animal models and human studies [27]; thus, subsequent studies should optimize formulations to enhance bioavailability.

## 5. Conclusions

In conclusion, this study successfully prepared purified recombinant poIFN-α and demonstrated its validity in controlling PEDV infection both in vitro and in vivo, indicating its potential as a novel antiviral agent for controlling PED in the swine industry. Future research will focus on elucidating the detailed molecular mechanisms underlying the antiviral activity of poIFN-α against PEDV. Exploring the potential of combining poIFN-α with existing vaccines or other antiviral drugs to achieve synergistic effects. These findings provide a novel strategy for the prevention and control of PEDV and offer a valuable theoretical foundation for the treatment of diseases caused by coronaviruses.

## Figures and Tables

**Table 1:** Primers used in this study.

Primers	Sequence (5′-3′)	Amplification Size (bp)
IFN-F	GCGGATCCGTGCGACCTGCCACAGACTC	509
IFN-R	GCAAGCTTTCATTCTTTCTTACGCAGACG	

## Data Availability

The original contributions presented in this study are included in the article/Supplementary Materials. Further inquiries can be directed to the corresponding authors.

## Supplementary Materials

The following are available online at https://www.mdpi.com/article/10.3390/vetsci13030249/s1, Figure S1: Original images of Figure 1, Figure S2: Original images of Figure 2.
