# Supplementary material for: Validation of Recombinant Type I Interferon Antiviral Activity Against Porcine Epidemic Diarrhea Virus In Vitro and In Vivo
Source: Vet Sci. 2026 Mar 6;13(3):249. doi: 10.3390/vetsci13030249 (PMC13029875; doi:10.3390/vetsci13030249)

Figure S2: Original images of Figure 2

Figure S2A-MOCK-2h

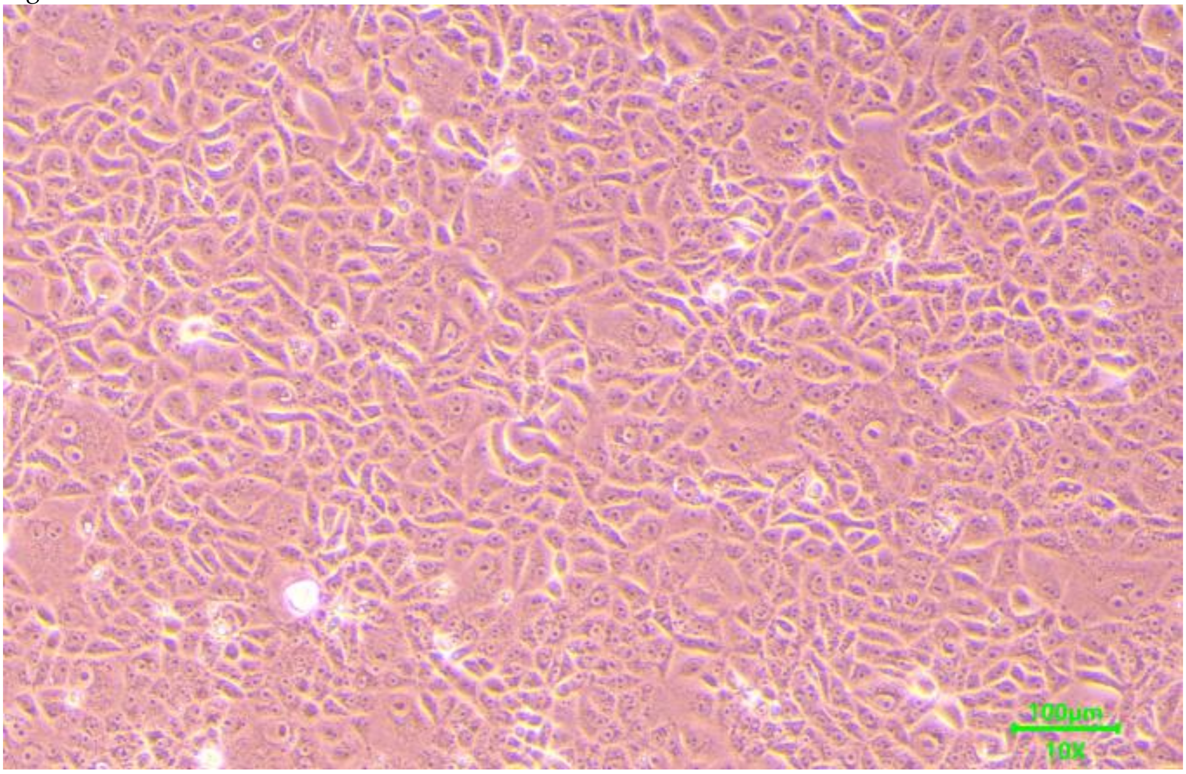

Figure S2A-MOCK-4h

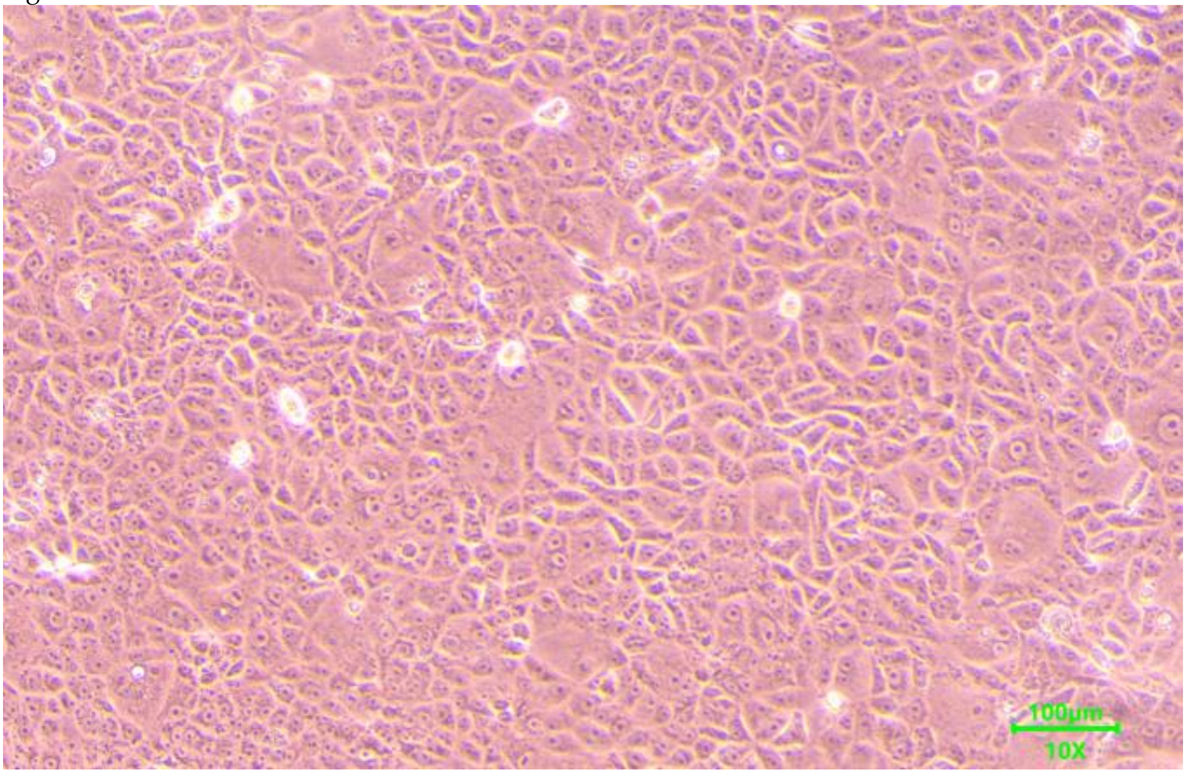

Figure S2A-MOCK-6h

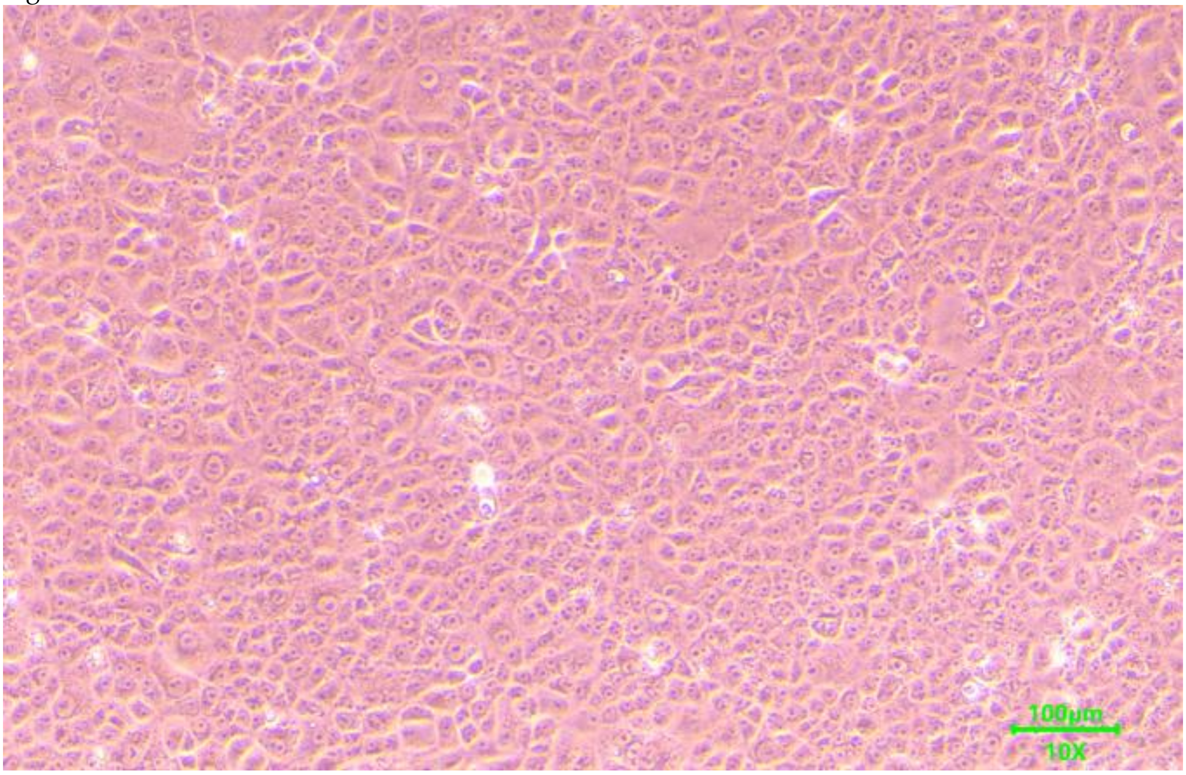

Figure S2A-MOCK-8h

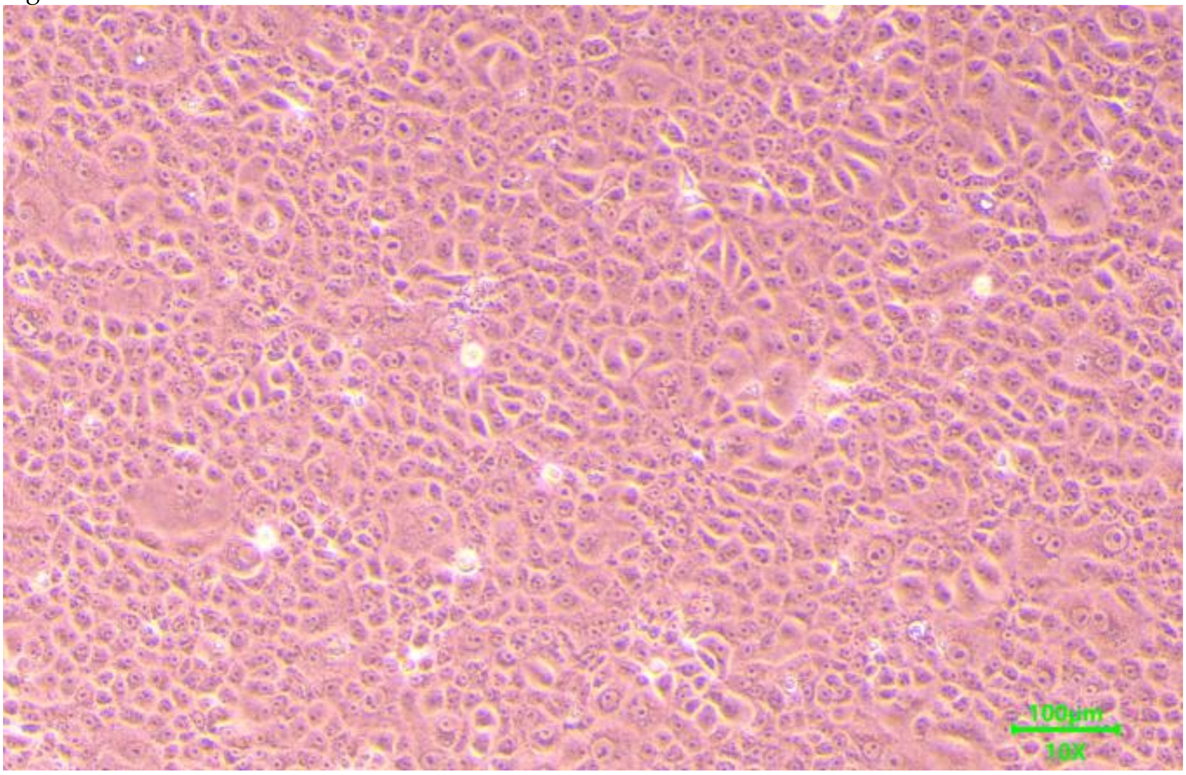

Figure S2A-MOCK-12h

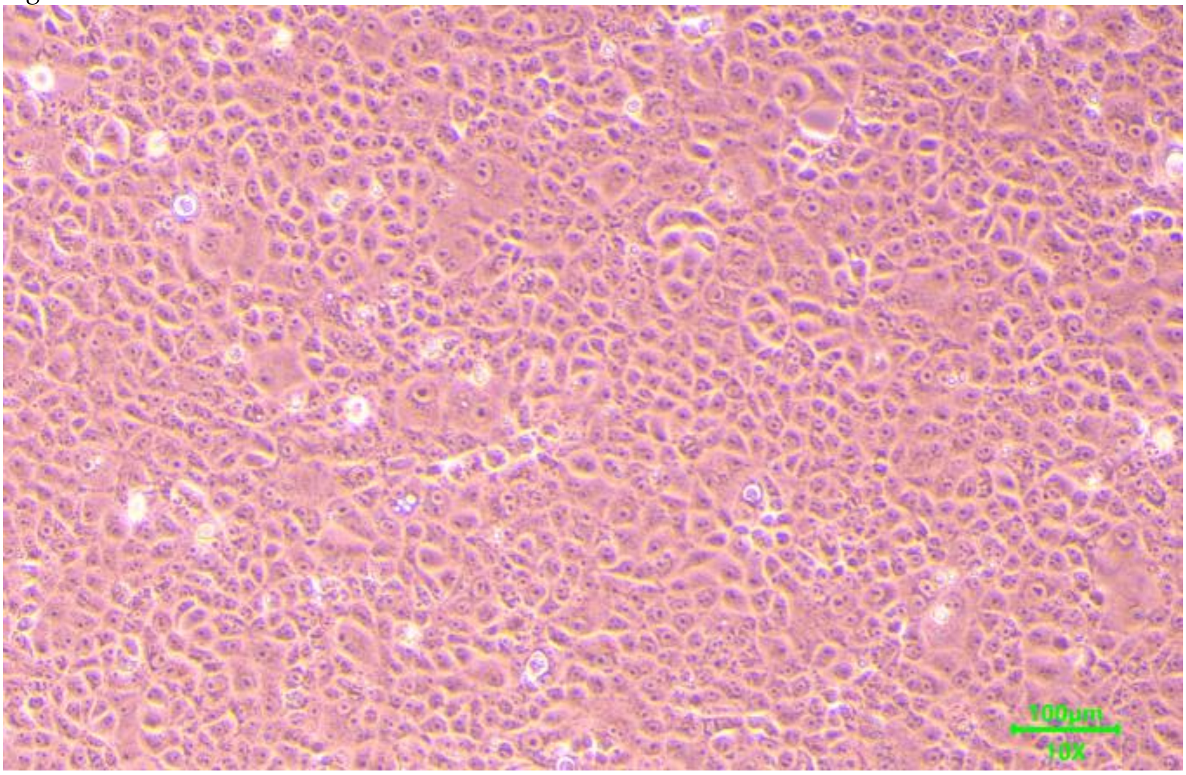

Figure S2A-MOCK-24h

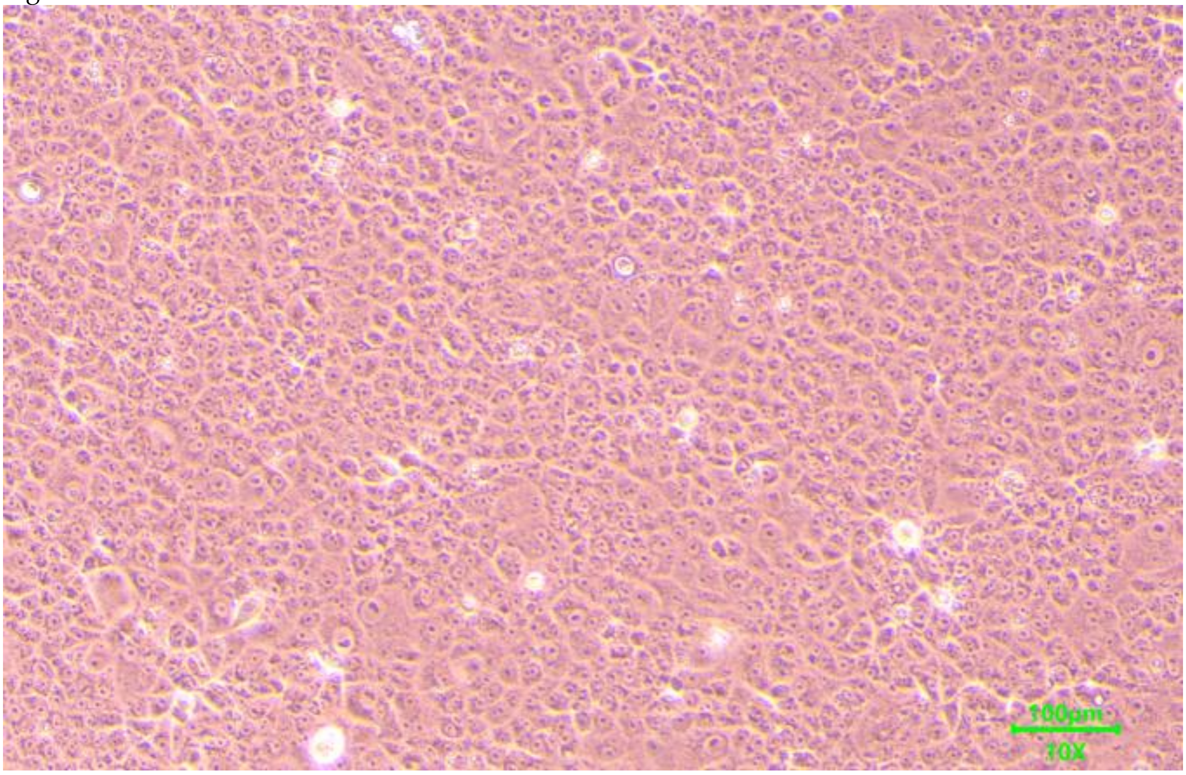

Figure S2A-MOCK-36h

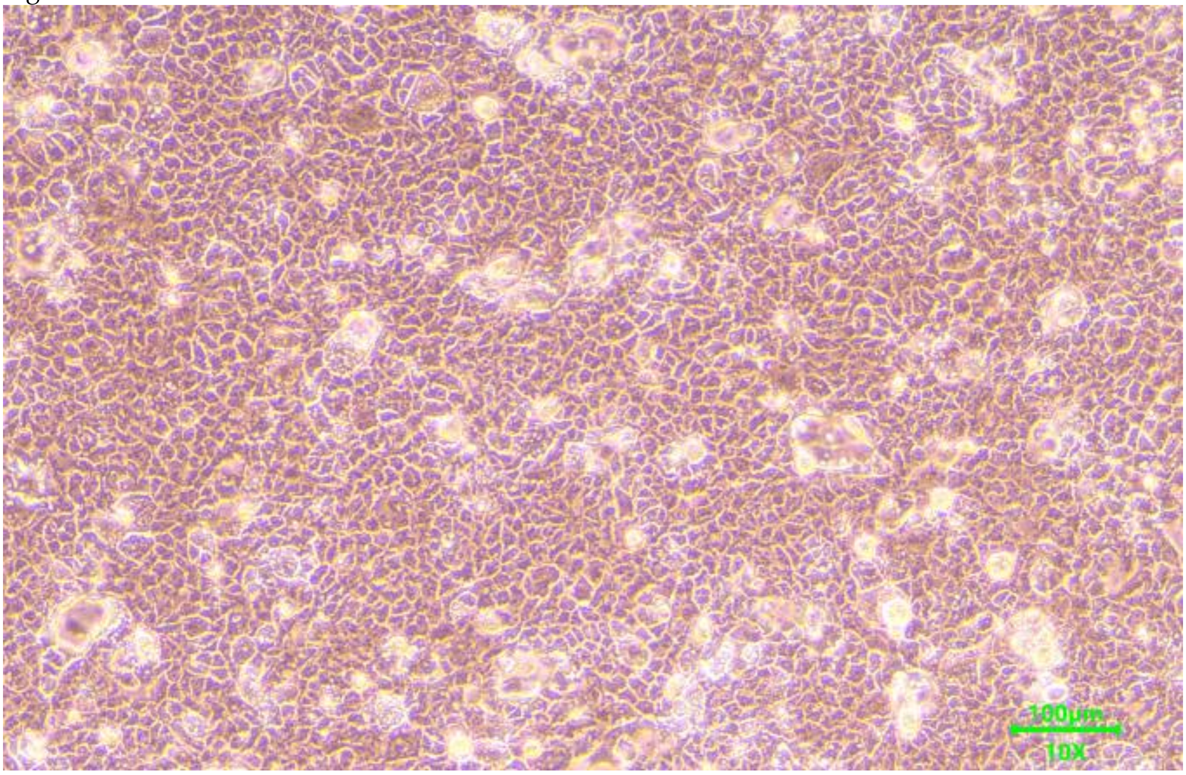

Figure S2A-PEDV-2h1

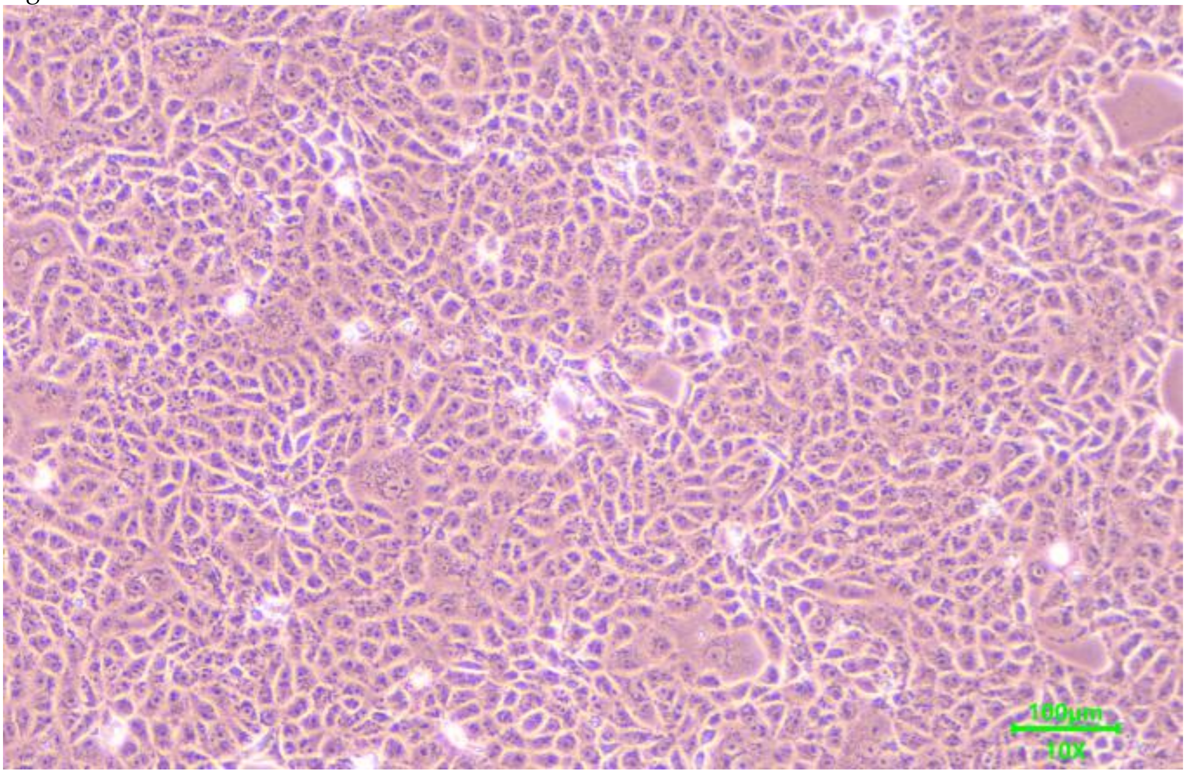

Figure S2A-PEDV-4h1

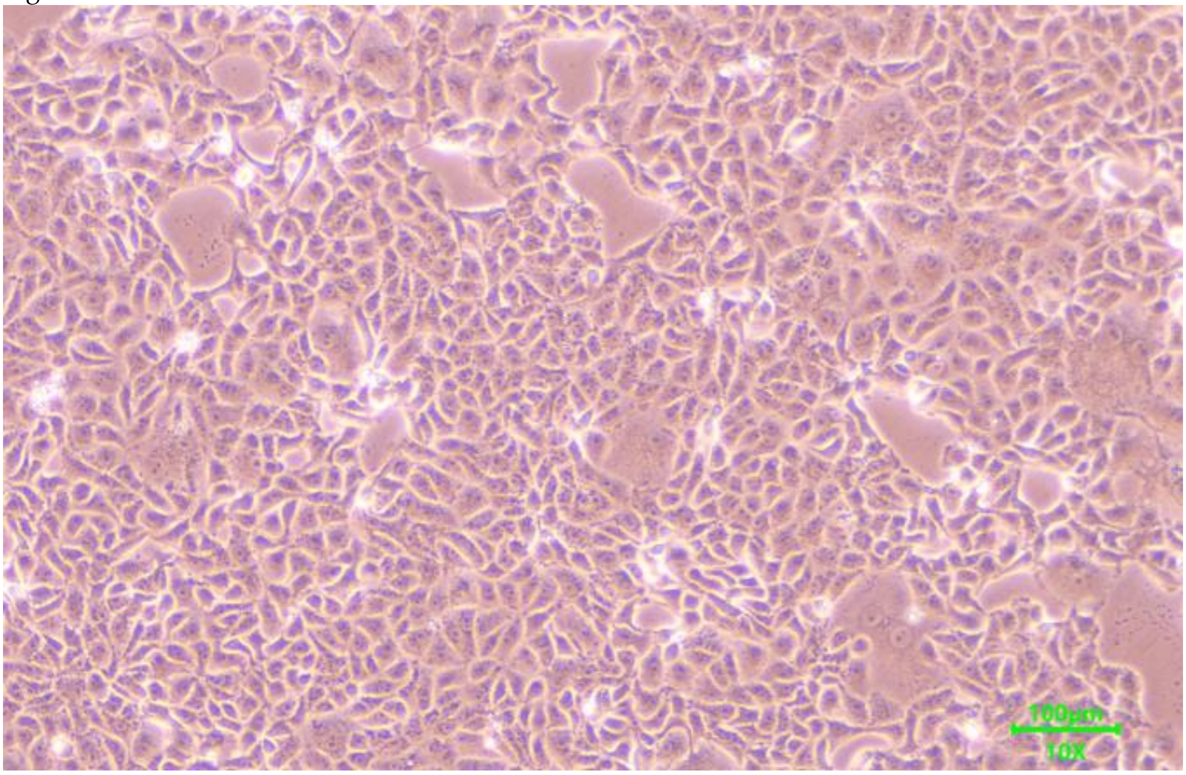

Figure S2A-PEDV-6h1

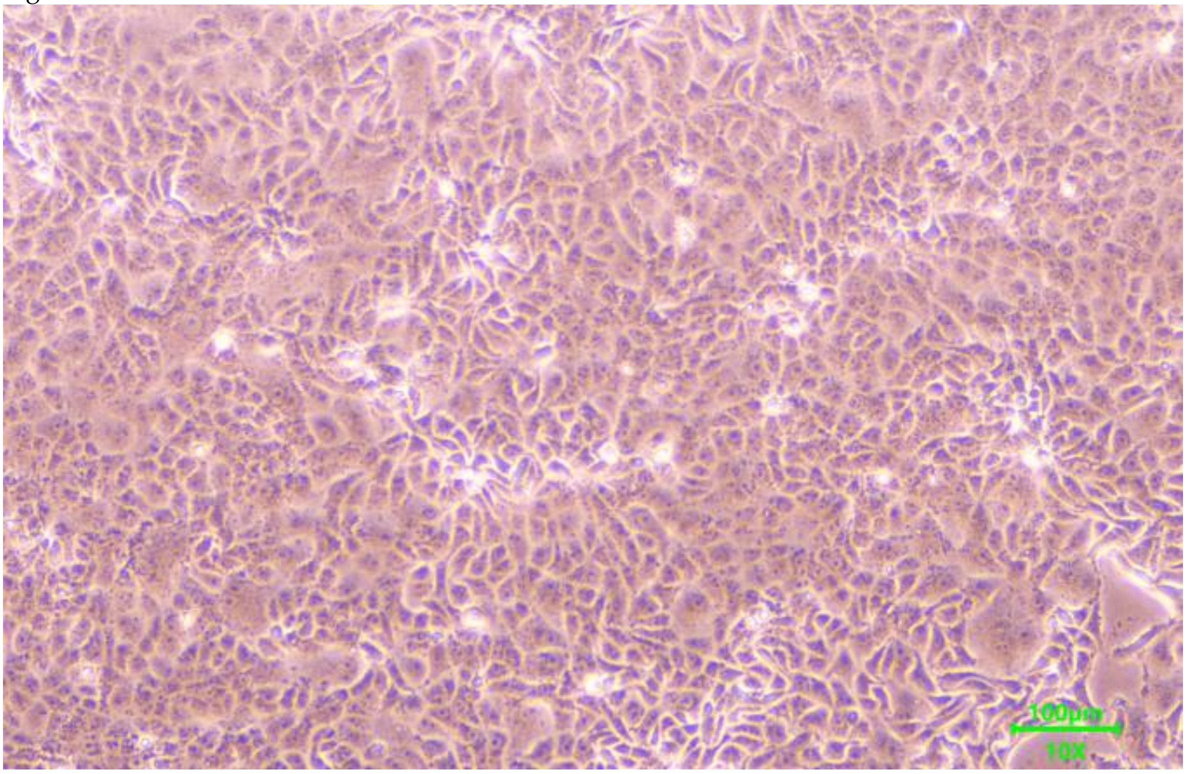

Figure S2A-PEDV-8h1

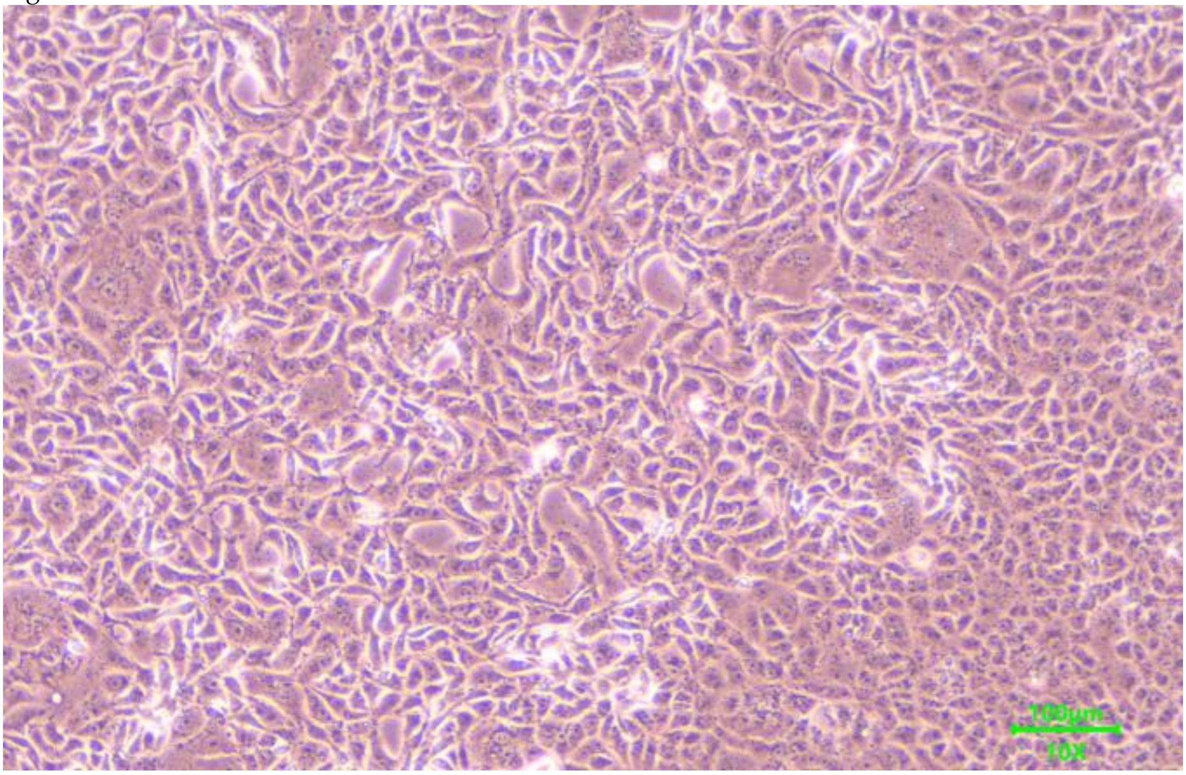

Figure S2A-PEDV-12h1

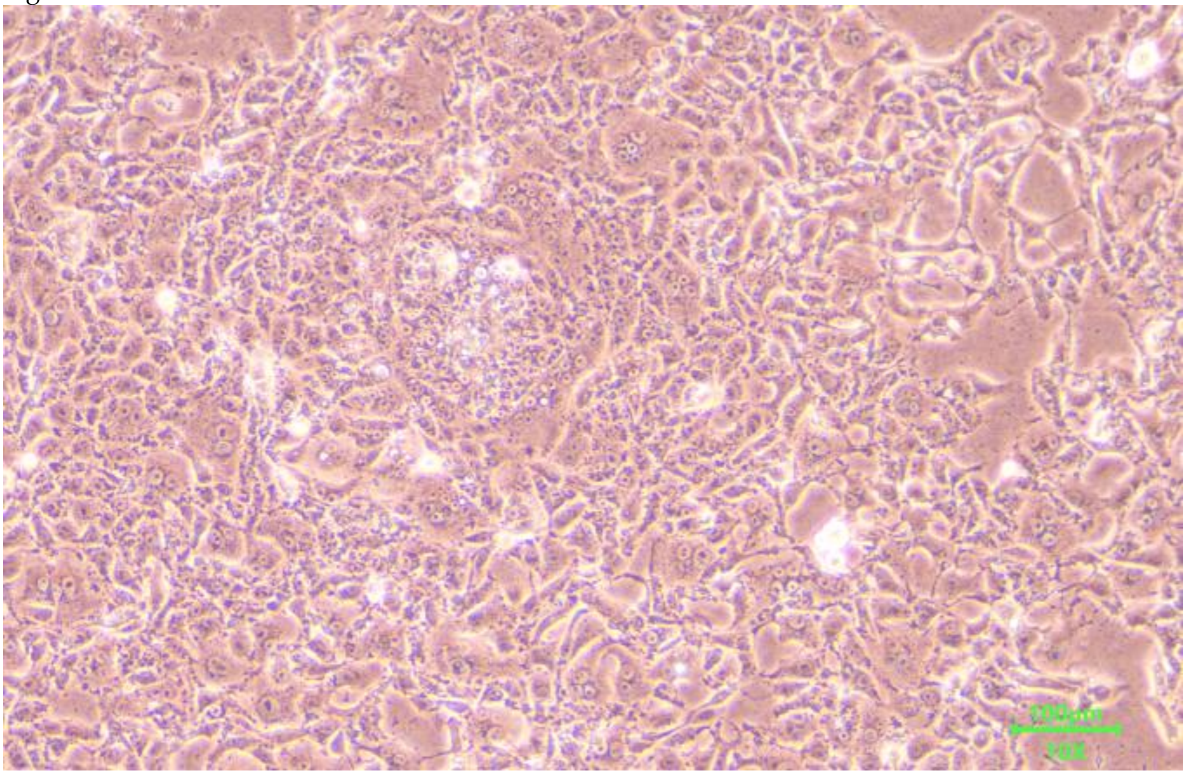

Figure S2A-PEDV-24h1

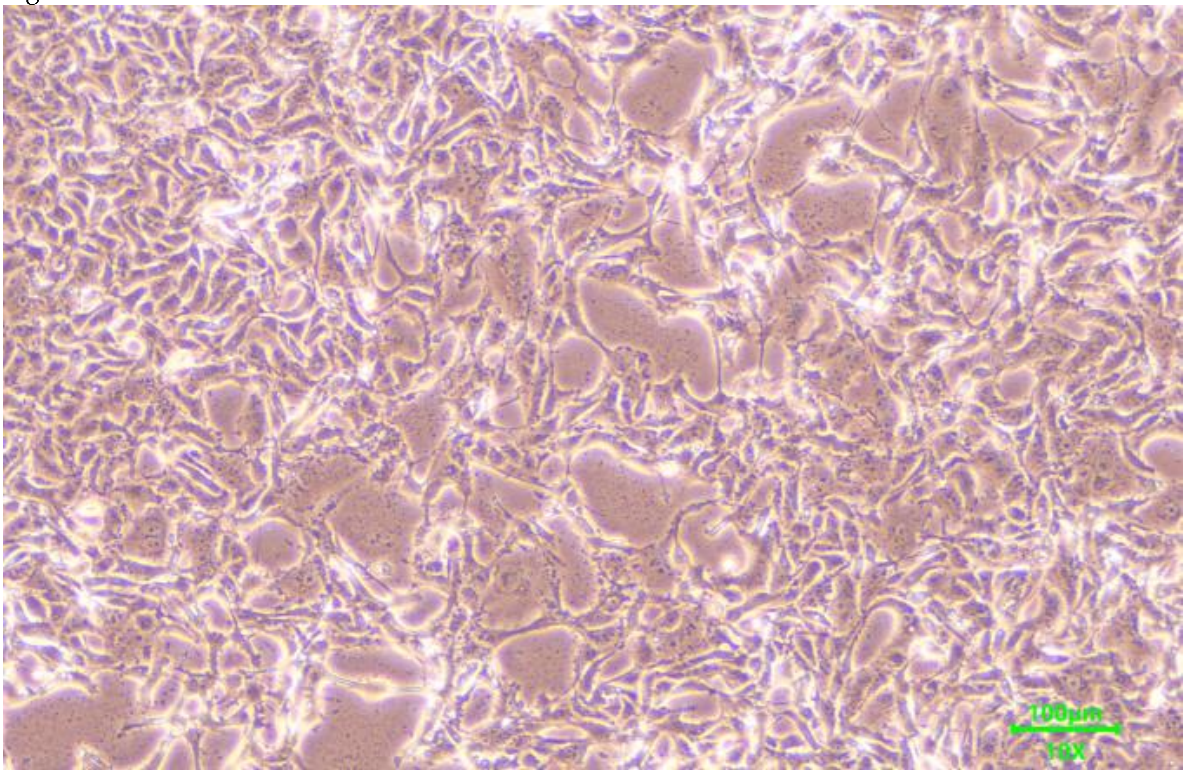

Figure S2A-PEDV-36h1

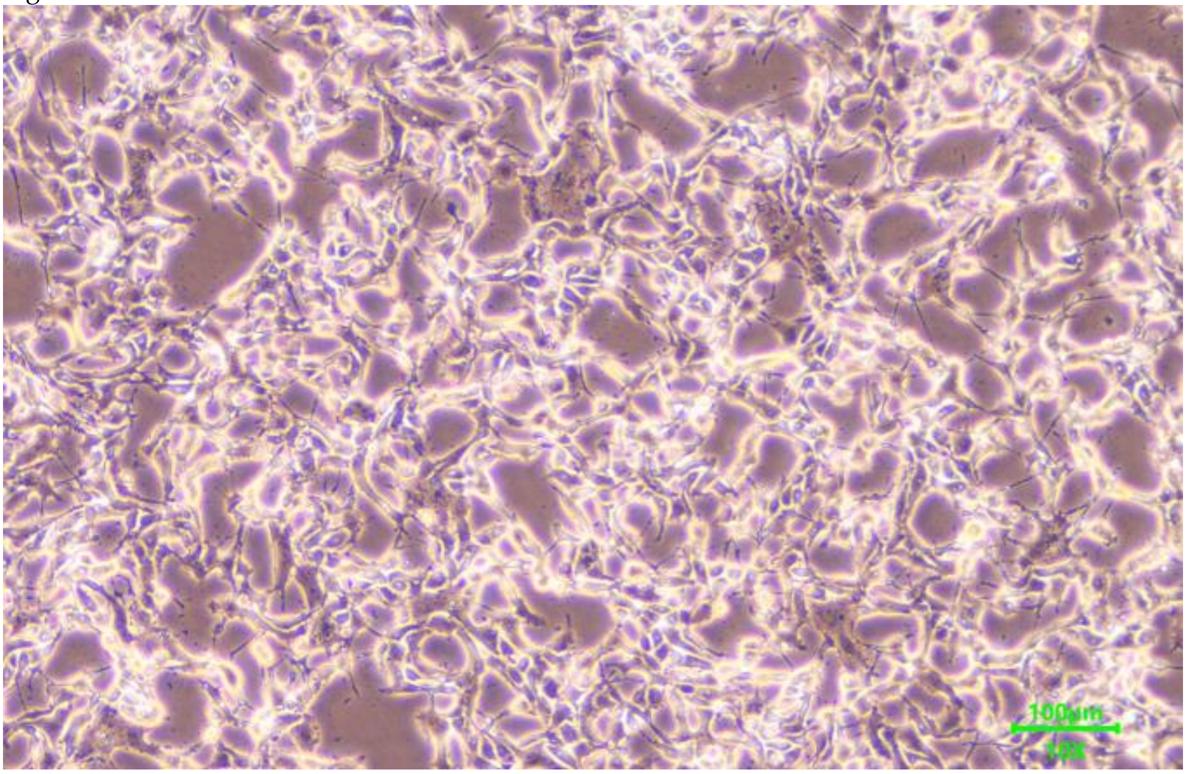

Figure S2A-PEDV+IFN-2h1

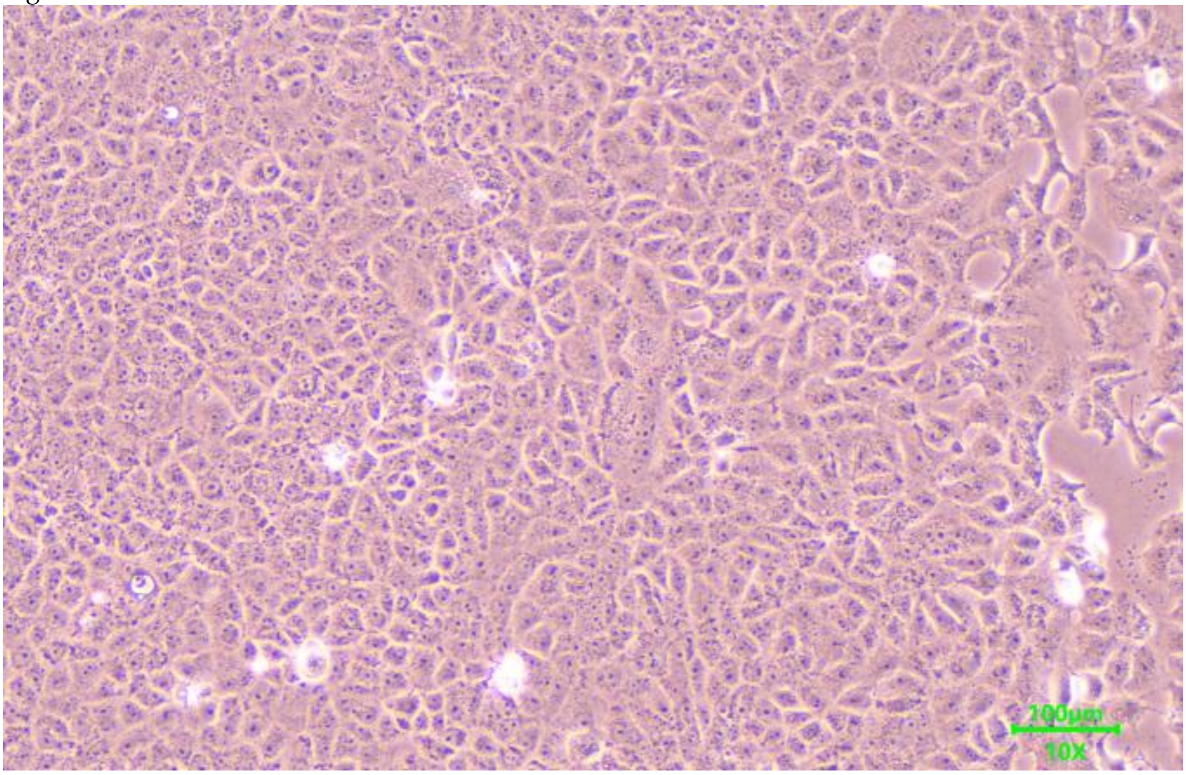

Figure S2A-PEDV+IFN-4h1

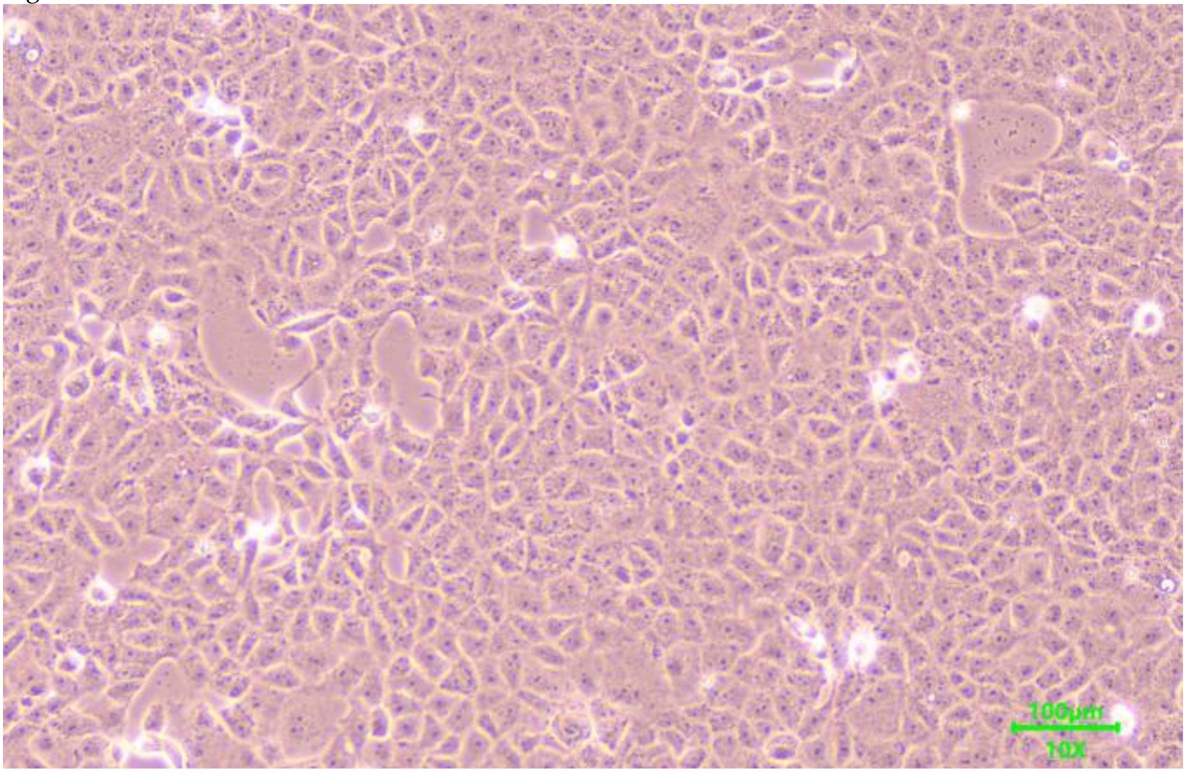

Figure S2A-PEDV+IFN-6h1

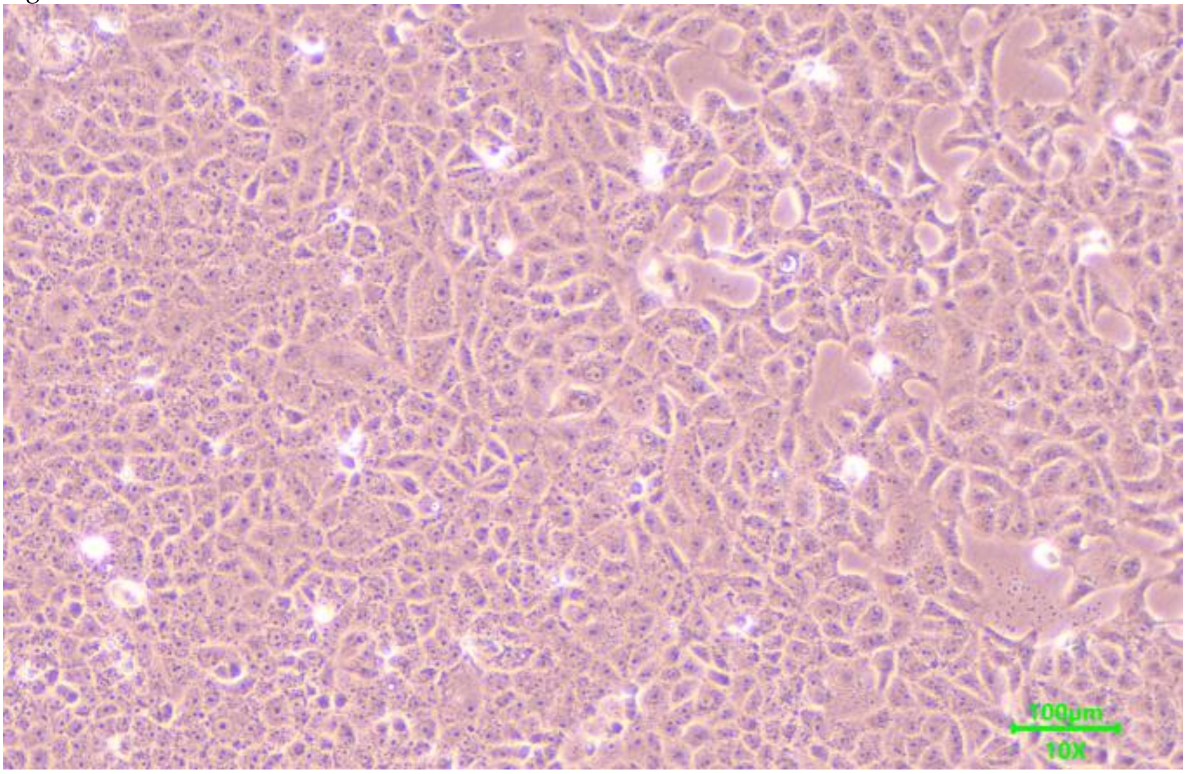

Figure S2A-PEDV+IFN-8h1

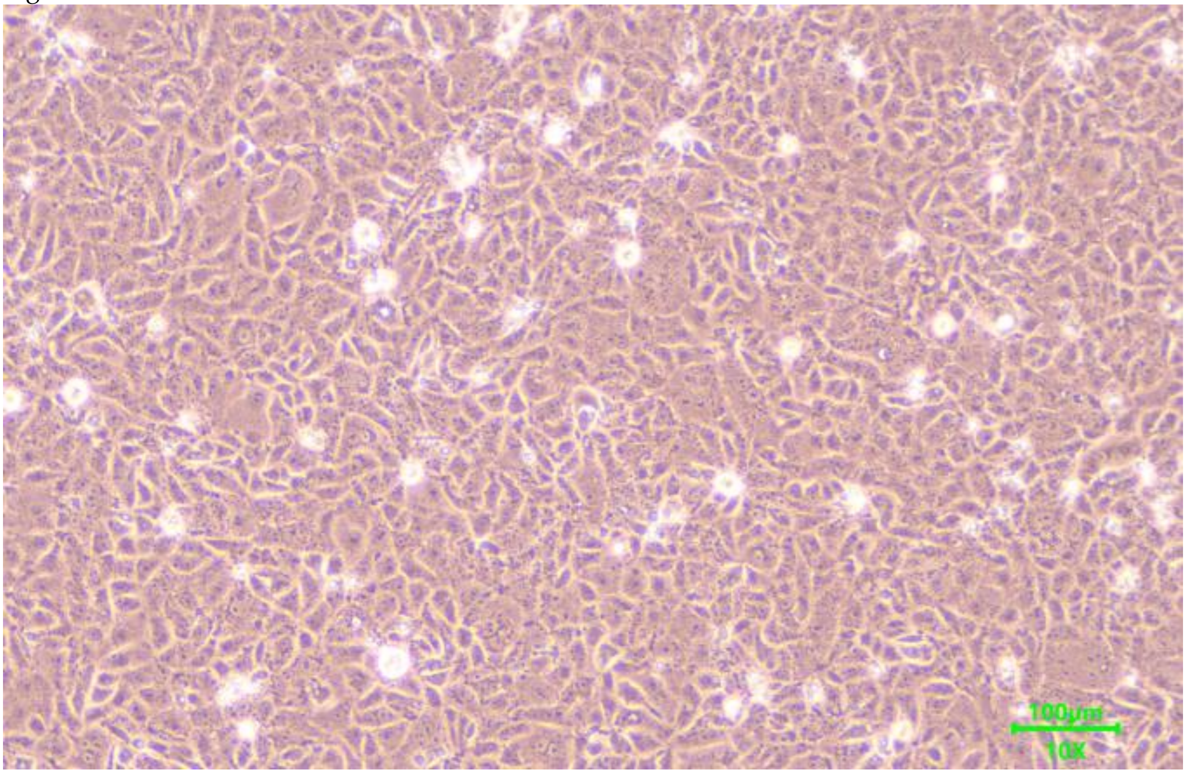

Figure S2A-PEDV+IFN-12h1

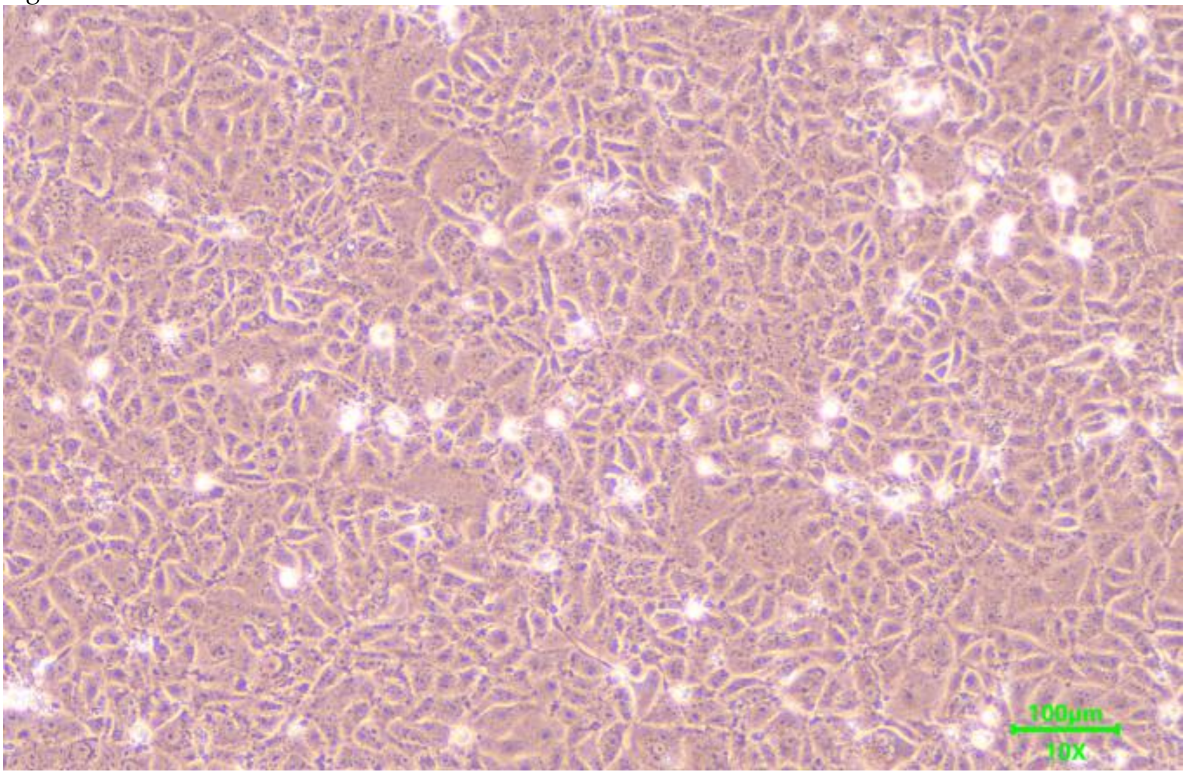

Figure S2A-PEDV+IFN-24h1

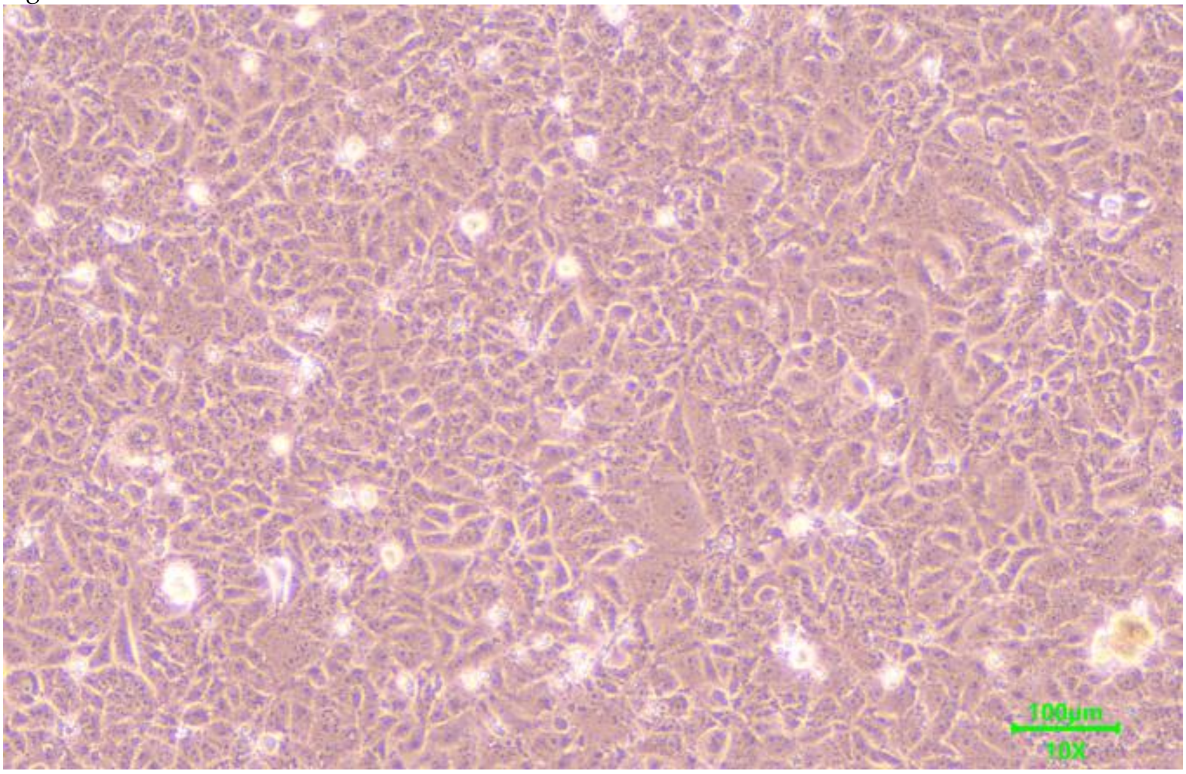

Figure S2A-PEDV+IFN-36h1

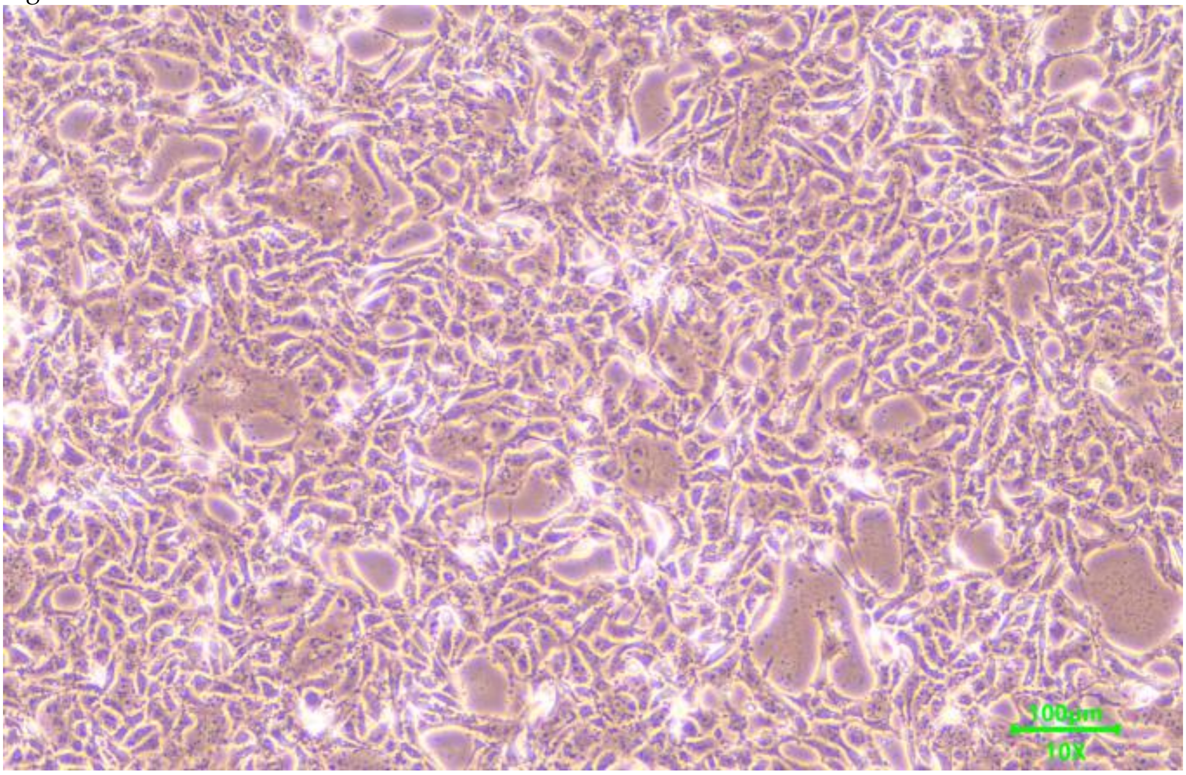

Supplement: Supplementary file 1 [file vetsci-13-00249-s001.zip › figure S2.pdf]
