# Supplementary figures and images for: Validation of Recombinant Type I Interferon Antiviral Activity Against Porcine Epidemic Diarrhea Virus In Vitro and In Vivo
Source: Vet Sci. 2026 Mar 6;13(3):249. doi: 10.3390/vetsci13030249 (PMC13029875; doi:10.3390/vetsci13030249)

Figure S1: Original images of Figure 1

A

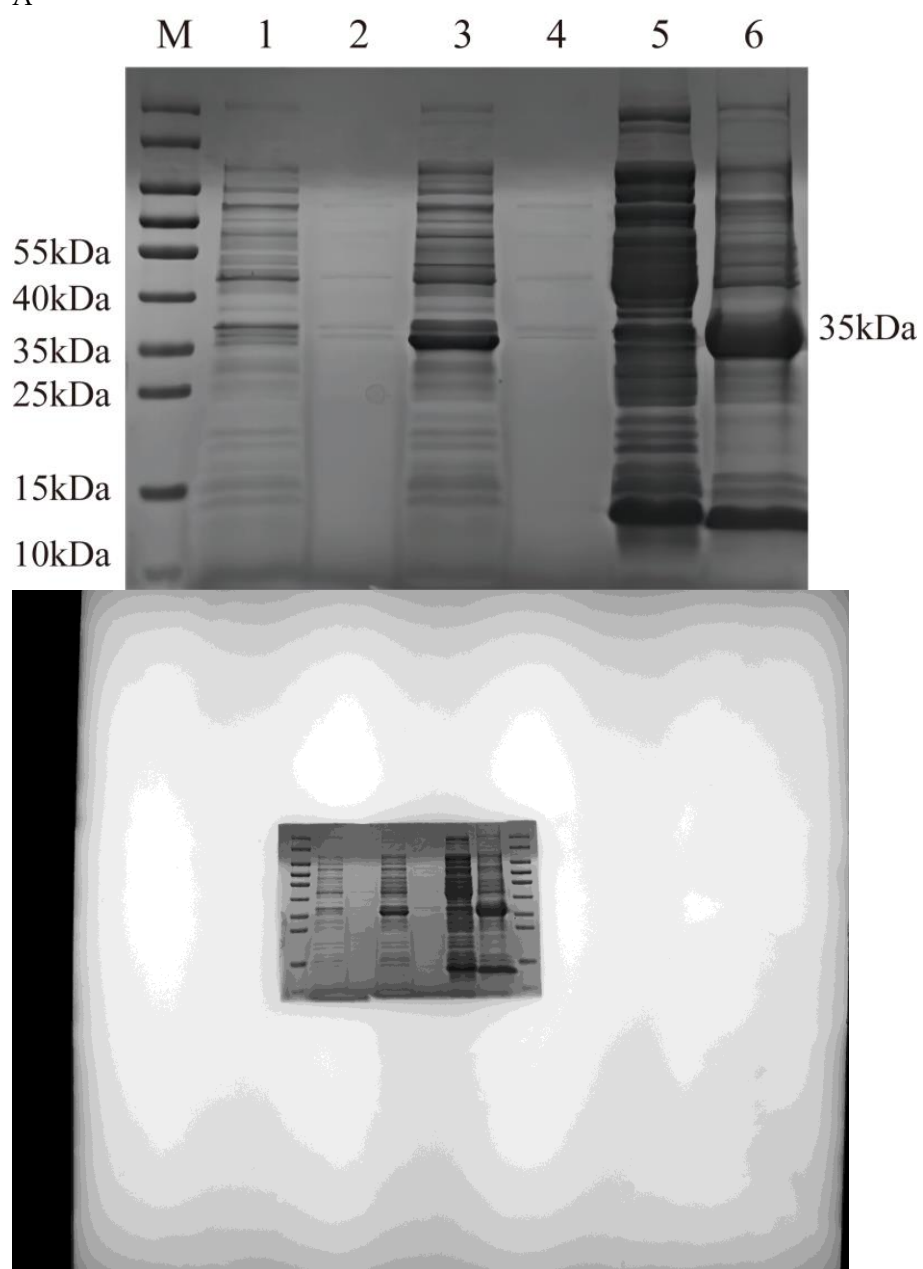

B

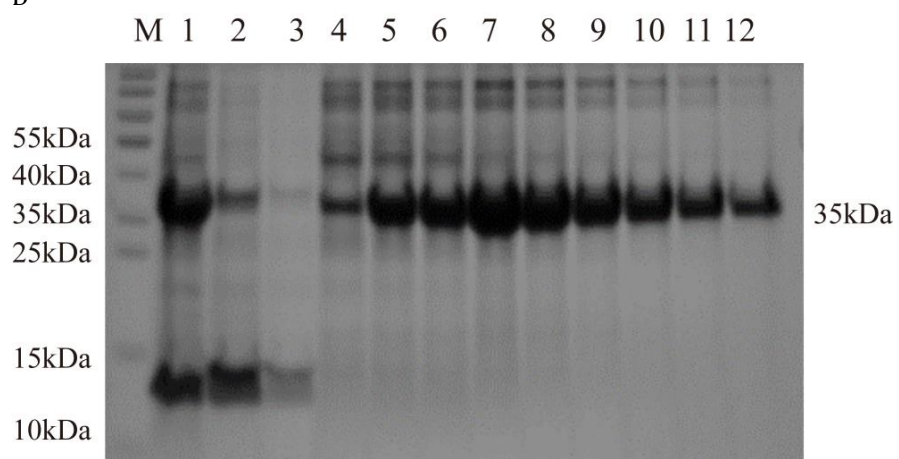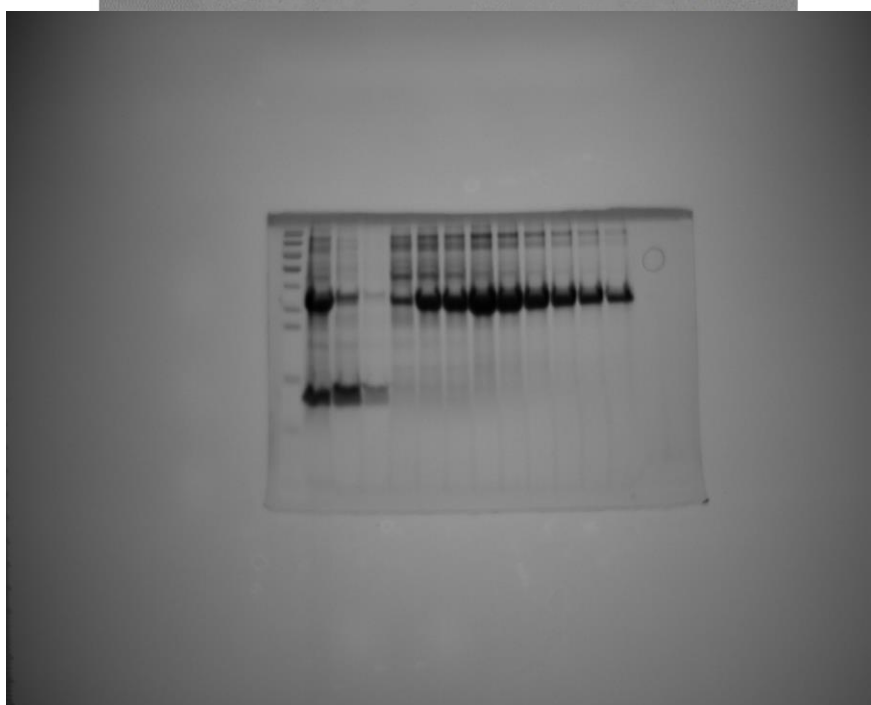

C

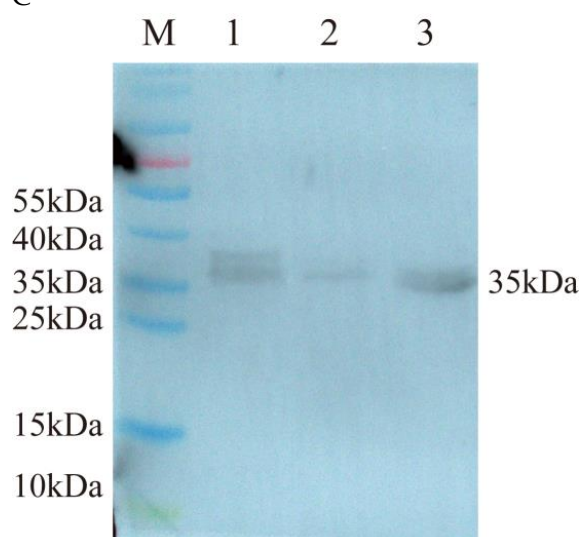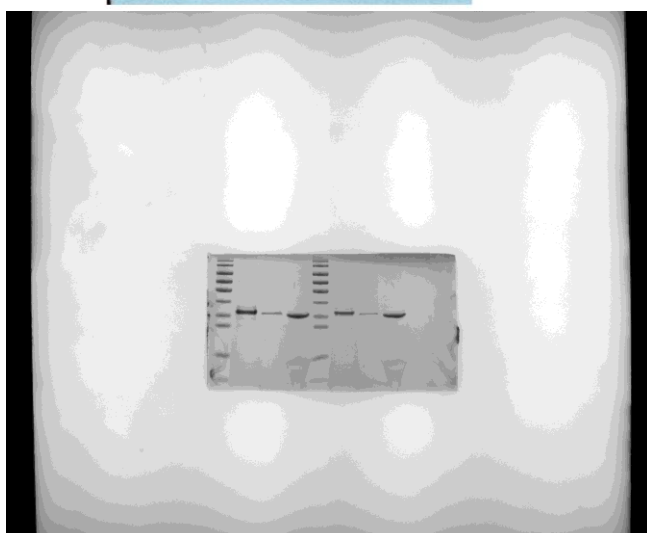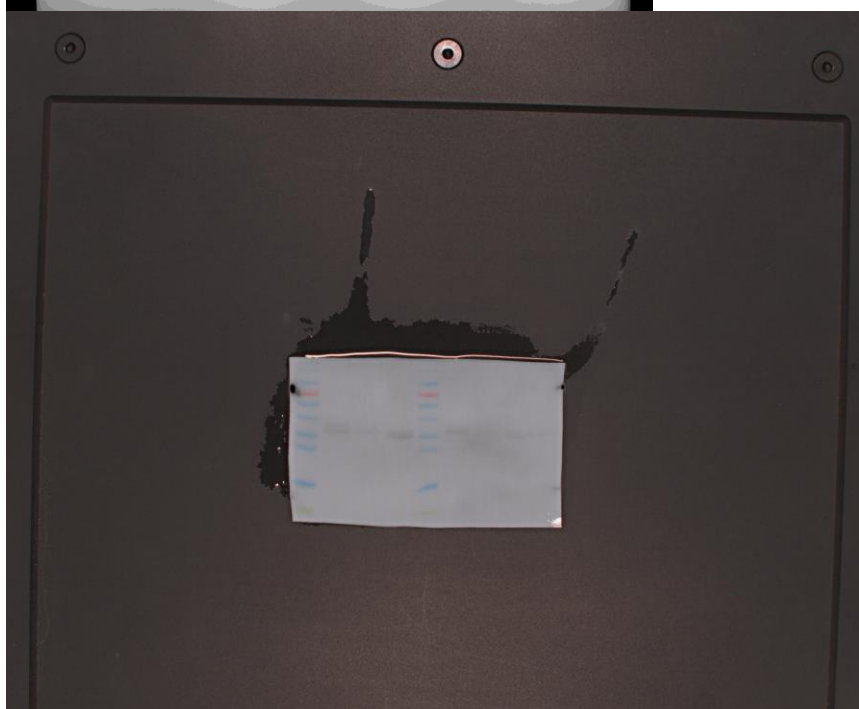

Supplement: Supplementary file 1 [file vetsci-13-00249-s001.zip › figure S1.pdf]
